# *CYP2C19* genotype testing for clopidogrel: A guideline developed by the UK Centre of Excellence in Regulatory Science and Innovation in Pharmacogenomics (CERSI‐PGx)

**DOI:** 10.1002/bcp.70370

**Published:** 2025-12-03

**Authors:** Cinzia Dello Russo, Iain Frater, Rebecca Kuruvilla, Stefanie Lip, Hannah O'Neill, Kerry Burke, Vicky Chaplin, Alexander S. F. Doney, Salim Elyas, Nicholas Greaves, Sophie Harding, David Hargroves, Judith Hayward, Dyfrig A. Hughes, Tom A. T. Hughes, Sree Kondapally, Patrick Mok, Aaron Peace, Imran Rafi, Simon Ray, Vicky Stinton, Luigi Venetucci, Munir Pirmohamed

**Affiliations:** ^1^ Department of Pharmacology and Therapeutics, Institute of Systems, Molecular and Integrative Biology University of Liverpool Liverpool UK; ^2^ Department of Healthcare Surveillance and Bioethics, Section of Pharmacology Università Cattolica del Sacro Cuore ‐ Fondazione Policlinico Universitario A. Gemelli, IRCCS Rome Italy; ^3^ School of Cardiovascular and Metabolic Health University of Glasgow Glasgow UK; ^4^ The Wolfson Centre for Personalised Medicine, Centre for Drug Safety Science University of Liverpool Liverpool UK; ^5^ Queen Elizabeth University Hospital Glasgow UK; ^6^ East of Scotland Vascular Network, Department of Vascular Surgery University of Dundee Dundee UK; ^7^ Department of Vascular Surgery, Manchester Royal Infirmary Manchester University NHS Foundation Trust Manchester UK; ^8^ Manchester Centre for Genomic Medicine St Mary's Hospital, Manchester University NHS Foundation Trust Manchester UK; ^9^ Division of Evolution, Infection and Genomics, School of Biological Sciences The University of Manchester Manchester UK; ^10^ Genomics Unit NHS England London UK; ^11^ School of Medicine University of Dundee Dundee UK; ^12^ The Academic Department of Healthcare for Older People Royal Devon University Healthcare NHS Foundation Trust Exeter UK; ^13^ Department of Clinical and Biomedical Science University of Exeter Medical School Exeter UK; ^14^ Velindre Cancer Centre Cardiff and Vale University Cardiff UK; ^15^ Department of Stroke Medicine East Kent Hospitals University NHS Foundation Trust Canterbury UK; ^16^ NW GMSA, NHSE Genomics Education Programme, Affinity Care London UK; ^17^ Centre for Health Economics and Medicines Evaluation, North Wales Medical School Bangor University Bangor UK; ^18^ Department of Neurology University Hospital of Wales Healthcare NHS Trust Cardiff UK; ^19^ Cardiovascular Clinical Academic Group, Molecular and Clinical Sciences Research Institute St. George's University of London London UK; ^20^ St. George's University Hospitals NHS Foundation Trust London UK; ^21^ Doncaster and Bassetlaw Teaching Hospitals NHS Foundation Trust Doncaster UK; ^22^ Western Health and Social Care Trust, Department of Cardiology and Clinical Translational Research and Innovation Centre Ulster University Londonderry UK; ^23^ St. George's University of London London UK; ^24^ Department of Cardiology Manchester University NHS Foundation Trust Manchester UK; ^25^ North West GLH The University of Manchester Manchester UK; ^26^ Faculty of Biology, Medicine and Health The University of Manchester Manchester UK

**Keywords:** clopidogrel, coronary artery disease, CYP2C19, health economics, peripheral arterial disease, pharmacogenetic testing, stroke

## Abstract

Clopidogrel, an antiplatelet agent, is currently licensed in the United Kingdom for the prevention and treatment of atherothrombotic events in cerebrovascular disease, coronary artery disease and peripheral arterial disease. Clopidogrel requires metabolic activation by the cytochrome P450 enzyme CYP2C19 to be effective. CYP2C19 is encoded by a polymorphic gene; variants in the *CYP2C19* gene, which vary in frequency in different ethnic groups can abolish, reduce or increase enzyme activity, thereby affecting the conversion of clopidogrel to its active metabolite. Individuals who have either one or two loss‐of‐function alleles are referred to as intermediate and poor metabolisers, respectively, and in these patients, the clinical effectiveness of clopidogrel is reduced or absent. Any patient about to be prescribed clopidogrel, regardless of the underlying indication, should have pharmacogenetic testing to identify clinically relevant *CYP2C19* variants, where testing is available, to optimize their antiplatelet therapy. Clopidogrel use should be avoided in patients with an intermediate or poor CYP2C19 metaboliser phenotype in all approved indications and alternative treatment regimens used as detailed in this guideline. Our guideline is compatible with other international pharmacogenetic prescribing guidelines, but we also provide recommendations in other areas. Summary guidance on a page is provided for each of the indications in Boxes 1–3. This guideline is grounded in the latest evidence in this field but cannot account for all individual factors relevant to patient care. Therefore, prescribers must conduct a thorough assessment of each patient's risk–benefit profile, ensuring that therapy is optimized to maximize benefits while minimizing potential harms.

## 1 BACKGROUND AND OVERVIEW

Clopidogrel is a commonly used antiplatelet (P2Y12 platelet inhibitor) agent for the prevention and treatment of atherothrombotic events [cerebrovascular disease, coronary artery disease (CAD) and peripheral arterial disease (PAD)].

Clopidogrel is a prodrug which requires enzymatic conversion into an active thiol metabolite. Only about 15% of clopidogrel is converted into the active H4 metabolite through a two‐step metabolic process in the liver, catalysed by multiple P450 isoforms, but predominantly CYP2C19. Variation in the activity of CYP2C19 can affect the formation of the thiol metabolite, potentially altering the clinical response to clopidogrel therapy. Genetic polymorphisms in *CYP2C19* are common and can lead to a number of phenotypes (see Table 1) which can decrease or increase enzyme activity or have no effect. The most common loss‐of‐function (LOF) alleles are CYP2C19*2 and *3 that result in degraded or nonfunctional proteins.^1^ The frequencies of these polymorphisms and hence the predicted phenotype vary according to ethnicity. For example, in European ancestry populations, the frequency of the LOF allele CYP2C19*2 is about 15%, while it is 30% in South Asians and 60% in native Oceanians.^2^ Conversely, CYP2C19*17 leads to increased protein expression and activity, but the clinical implications of this are unclear.^3^

**TABLE 1:** CYP2C19 phenotypes based on tier 1 *CYP2C19* allelic variants.

*CYP2C19* diplotype	Change in CYP2C19 activity	Predicted phenotype
*17/*17	*17 allele increases protein expression and metabolic activity	Ultra‐rapid metaboliser
*1/*17	*1 is a normal function allele	Rapid metaboliser
*1/*1	*1 is a normal function allele	Normal metaboliser
*1/*2, *1/*3, *2/*17, *3/17	Both *2 and *3 are no function alleles.	Intermediate metaboliser
*2/*2, *3/*3, *2/*3	Both *2 and *3 are no function alleles	Poor metaboliser

CYP2C19 can also be inhibited by concomitantly administered drugs, for example omeprazole, effectively reducing the catalytic activity of the enzyme, a process called phenoconversion (this is discussed later).

## 2 LICENSED INDICATIONS

**Table:** 

**Currently licensed indications for clopidogrel in the UK** ^**4**^ *
**A. *Secondary prevention of atherothrombotic events*** Clopidogrel is indicated in: Adult patients suffering from myocardial infarction (from a few days until less than 35 days), ischaemic stroke (from 7 days until less than 6 months) or established PAD. Adult patients suffering from acute coronary syndrome: Non‐ST segment elevation acute coronary syndrome (unstable angina or non‐Q‐wave myocardial infarction), including patients undergoing a stent placement following percutaneous coronary intervention, in combination with acetylsalicylic acid (ASA). ST segment elevation acute myocardial infarction, in combination with ASA in patients undergoing percutaneous coronary intervention (including patients undergoing a stent placement) or in medically treated patients eligible for thrombolytic/fibrinolytic therapy. **B. *In patients with moderate to high‐risk Transient Ischemic Attack (TIA) or minor Ischemic Stroke (IS)*** Clopidogrel in combination with ASA is indicated in: Adult patients with moderate to high‐risk TIA (ABCD2 score ≥ 4) or minor IS (NIHSS ≤ 3) within 24 h of either the TIA or IS event. **C. *Prevention of atherothrombotic and thromboembolic events in atrial fibrillation*** In adult patients with atrial fibrillation who have at least one risk factor for vascular events, are not suitable for treatment with Vitamin K antagonists (VKA) and who have a low bleeding risk, clopidogrel is indicated in combination with ASA for the prevention of atherothrombotic and thromboembolic events, including stroke.

*The exact wording for the currently licensed indications has been extracted from the Summary of Product Characteristics of Plavix, approved by the Medicines Healthcare products Regulatory Agency (MHRA, text revised on 15/12/2023).^4^ In this document, aspirin is reported as acetylsalicylic acid (ASA).

It is important to note that routine prescribing of clopidogrel has changed over time within clinical practice because of various factors including (a) the introduction of alternative antiplatelet agents, (b) the introduction of direct oral anticoagulants and (c) changes in clinical practice. Clopidogrel is also used as a therapeutic option in the long‐term (often longer than 6 months) secondary prevention of atherothrombotic events, which is consistent with some of the national guidelines.^5^

## 3 EVIDENCE OVERVIEW

### 3.1 Stroke and transient ischaemic attack

A retrospective study of the CHANCE trial population in China showed that the benefit of dual antiplatelet therapy (DAPT) with aspirin plus clopidogrel *vs*. aspirin monotherapy was absent in intermediate or poor metabolisers with LOF alleles in *CYP2C19* (9.4% *vs*. 10.8%; hazard ratio, HR: 0.93, 95% confidence interval, 95% CI: 0.69–1.26; *p* = .64).^6^ CHANCE 2 was a large double‐blind, placebo controlled randomized study also in a Han‐Chinese population with transient ischaemic attack (TIA) or minor stroke, where patients were allocated to ticagrelor or clopidogrel alongside aspirin within 24 h of symptom onset, all of whom had a *CYP2C19* LOF allele. The risk of stroke was reduced from 7.6% in patients on clopidogrel to 6% in the ticagrelor group (HR: 0.77; 95% CI: 0.64–0.94; *p* = .008).^7^ A subgroup analysis per genotype showed similar efficacy of ticagrelor in both *CYP2C19* intermediate and poor metaboliser groups. More bleeding events were observed in the ticagrelor group (5.3% *vs*. 2.5%, HR: 2.18; 95% CI: 1.66–2.85), although the incidence of severe or moderate bleeding was similar in the two groups (0.3%).^7^

There have been several recent meta‐analyses summarizing the effects of *CYP2C19* LOF alleles on the risk of recurrent vascular events. Pan et al. conducted a meta‐analysis of 15 studies in 4762 stroke or TIA patients from all ethnic groups with LOF alleles (*2, *3 and *8) treated with clopidogrel and found an increased risk of stroke compared to individuals without any LOF alleles (12% *vs*. 5.8%; risk ratio, RR: 1.92, 95% CI: 1.57–2.35; *p* < .001).^8^

Biswas et al. conducted a large meta‐analysis looking at endpoints of recurrent stroke, composite vascular events and bleeding. They included 28 studies (22 observational studies and 6 randomized clinical trials, RCTs) with 9443 stroke or TIA patients. They found that patients taking clopidogrel in the LOF allele group showed an increased risk of recurrent stroke compared with those with no LOF alleles (OR: 2.18, 96% CI: 1.80–2.63; *p* < .00001).^9^ Cargnin et al. performed a focused meta‐analysis of stroke and TIA patients of non‐East Asian ethnicity; they included eight studies with 1673 patients. Their analysis found a significant increase in recurrent stroke in the LOF allele groups treated with clopidogrel when compared with subjects who were normal metabolisers (RR: 1.68, 95% CI: 1.04–2.71; *p* = .03). This was also found in European patients with *CYP2C19* LOF alleles (RR: 2.69, 95% CI: 1.11–6.51, *p* = .03).^10^

Maas et al. performed a large meta‐analysis on clopidogrel and *CYP2C19* genotype status in all atherothrombotic events. Their analysis included 29 stroke studies and found that subjects with LOF alleles carried a higher risk of major adverse cardiovascular events (RR: 1.61, 95% CI: 1.25–2.08) and recurrent ischaemic stroke compared with *CYP2C19* wild‐type subjects (RR: 1.89, 95% CI: 1.48–2.40).^11^

The NICE diagnostic guidance undertook a meta‐analysis of 25 studies that showed that patients with LOF alleles are at increased risk of secondary vascular events (HR: 1.72, 95% CI: 1.43–2.08; 18 studies) and ischaemic stroke (HR: 1.99, 95% CI: 1.49–2.64; 12 studies) when treated with clopidogrel alone or in addition to short‐term aspirin compared with patients without LOF alleles.^12^ Meta‐regression did not show any difference in incidence of vaso‐occlusive end points for these studies for location (China, Europe, Asia non‐China, Turkey and International) or different ethnicities (Asian or mixed to white). The authors did an analysis of seven RCTs comparing alternative antiplatelet therapies in LOF allele populations with stroke/TIA. There is a suggestion from two studies that ticagrelor was associated with a reduced risk of secondary vascular events (summary HR: 0.76, 95% CI: 0.65–0.90), albeit with a slightly increased risk of bleeding.^12^ The NICE diagnostic guidance in 2024 has demonstrated that *CYP2C19* testing for new non‐minor ischaemic stroke and TIA was both clinically effective and cost effective.^13^ The 2023 National Clinical Guideline for stroke for the United Kingdom and Ireland^5^ recommended testing after recurrent events while taking clopidogrel.

### 3.2 CAD

Upfront use of pharmacogenetic testing for clopidogrel is not currently routine clinical practice in the United Kingdom for the management of individuals with CAD.^14^ While this could be driven by the use of more potent P2Y12 inhibitors as part of DAPT regimens (such as aspirin + ticagrelor or aspirin + prasugrel), the underutilization may also reflect the high success (and low complication) rates of contemporary percutaneous coronary intervention (PCI), as well as a lack of awareness among physicians as to the role of routine pharmacogenetic testing. Against this backdrop, in 2024, the American Heart Association released a statement supporting clopidogrel pharmacogenetic testing when used for cardiovascular disease.^15^ Their analysis included a summary of meta‐analyses, which all point to subjects with *CYP2C19* LOF alleles experiencing more major adverse cardiovascular events (MACE).

Of the clinical trials reviewing outcomes with genotype guided care, the TAILOR‐PCI and POPular Genetics trials had the largest cohorts. In the TAILOR‐PCI trial^16^ the primary endpoint, a composite of cardiovascular death, myocardial infarction, stroke, stent thrombosis and severe recurrent ischemia at 12 months occurred in 4% of the genotype‐guided group (where patients with the *2 and *3 LOF variants were prescribed ticagrelor instead of clopidogrel) and in 5.9% of the conventional therapy group (HR: 0.66, 95% CI: 0.43–1.02; *p* = .06). A further analysis of the TAILOR‐PCI trial which captured both the first ischaemic event and subsequent events showed a reduction in the cumulative primary endpoint in the genotype‐guided group *vs*. the conventional therapy group (HR: 0.61, 95% CI: 0.41–0.89; *p* = .011).^17^ Importantly, in both the primary and the subsequent analyses, there was no evidence of an increase in minor or major bleeding from genotype guided care. The POPular Genetics trial showed that the genotype‐guided strategy was non‐inferior to standard treatment with ticagrelor or prasugrel, with a reduction in bleeding (HR: 0.78, 95% CI: 0.61–0.98; *p* = .04) in the genotype‐guided group, reinforcing the safety of genotype guided care.^18^

Following this, a 2022 meta‐analysis that included the results of the TAILOR‐PCI trial and POPular Genetics trial (alongside others) demonstrated a statistically significant reduction in MACE post‐PCI with genotype guided care (RR: 0.60, 95% CI: 0.44–0.82, *p* = .001, *I* ^2^ = 67%).^19^

### 3.3 PAD

Clopidogrel is licensed for PAD in the United Kingdom and remains first line therapy for secondary prevention of PAD, consistent with the guidelines from Vascular Society of Great Britain & Ireland (VSGBI)^20^ and NICE.^21^ It is accepted that the evidence base for the use of clopidogrel in PAD is not as extensive as that for stroke and coronary disease. However, it is also important to note that patients with PAD are likely to have atherosclerotic arterial disease in both the coronary and cerebral circulations, and hence, the use of clopidogrel will protect patients from atherothrombosis in both the peripheral and central arterial circulations.

In a systematic review of patients undergoing *CYP2C19* pharmacogenetic testing, 44 studies were included (CAD, Stroke and PAD), with just four relating to PAD.^11^ Of these studies, most of the included patients had undergone an intervention. While this review concluded that no meaningful conclusion could be drawn with regard to PAD outcomes, it was noted that patients with LOF alleles who had undergone endovascular therapy had a higher rate of restenosis.

This is complemented by Huang et al. who carried out a systematic review of four observational studies (sample sizes from 50 to 278) which showed that the presence of LOF alleles was associated with reduced clinical effectiveness of clopidogrel. However, they noted that the evidence base was limited and recommended the need for randomized controlled trials in PAD patients.^22^

## 4 RECOMMENDED INDICATIONS FOR PHARMACOGENETIC TESTING

Any patient who is about to be prescribed clopidogrel, regardless of the underlying indication, should have pharmacogenetic testing to identify clinically relevant *CYP2C19* variants, where testing is available, to optimize their antiplatelet therapy. Such pharmacogenetic information may already be available in their medical record, and if not, consideration should be given to obtaining it through pharmacogenetic testing.

Some patients already on clopidogrel may either have another atherothrombotic event or are at high risk of recurrent events. In such cases, consideration should be given to *CYP2C19* pharmacogenetic testing, if the information is not already available in the medical record.

The availability of pharmacogenetic testing may vary in time, indication and in geography. In the absence of available relevant pharmacogenetic information or testing, the current best practice clinical guidelines should be followed.

## 5 INTEGRATING PHARMACOGENETIC TESTING INTO EXISTING CLINICAL PATHWAYS

### 5.1 Stroke and transient ischaemic attack

#### 5.1.1 Minor ischaemic stroke and TIA

Pharmacogenetic testing for *CYP2C19* should be done when a diagnosis of TIA or minor ischemic stroke is made and treatment with clopidogrel is considered. Treatment with antiplatelets should not be delayed while waiting for the genotyping result. Local guidelines for antiplatelet therapy should be followed while awaiting *CYP2C19* results. Clopidogrel prescription may need to be changed in those who are either intermediate metabolisers or poor metabolisers (Table 1).

#### 5.1.2 Non‐minor ischaemic stroke

Pharmacogenetic testing for *CYP2C19* should be done at the time of stroke after haemorrhage has been excluded by CT scanning and in the absence of an immediate cardioembolic cause. Patients with non‐minor ischaemic stroke should receive aspirin for 2 weeks, after which clopidogrel (or other anti‐platelet agents, depending on the pharmacogenetic test result; see below) should be started.

### 5.2 CAD

Pharmacogenetic testing for *CYP2C19* variants in people with CAD should take into the account: (a) acuity of clinical presentation, (b) concomitant use of oral anticoagulants (OACs) and (c) risk of bleeding and recurrent CVD events in the individuals treated with clopidogrel. In the context of acute coronary syndromes, clopidogrel pharmacogenetic testing is recommended at the time of hospital admission for those individuals in whom clopidogrel is deemed to be the more suitable alternative to other potent P2Y12 inhibitors (due to high bleeding risk or concomitant use of OACs). In individuals diagnosed with chronic coronary syndromes (CCS, previously known as ‘stable CAD’), clopidogrel pharmacogenetic testing is recommended upon first contact with a specialist centre involved in either outpatient or day‐case management of these individuals (e.g., individuals attending cardiac catheterisation procedures). Regardless of the clinical context, initiation of clopidogrel (if deemed appropriate) should not be delayed following the first contact but must be modified based on the results of the genetic test.

### 5.3 PAD

PAD technically refers to areas beyond the lower limbs, including carotid and upper limb disease. Disease affecting the lower limbs is now termed lower extremity arterial disease (LEAD).

For lower limb ischaemia, there are three broad clinical scenarios:

1. Symptomatic LEAD (e.g., intermittent claudication).
  Pharmacogenetic testing for *CYP2C19* variants in people with LEAD should be undertaken at the time of diagnosis with aspirin being commenced while waiting for the *CYP2C19* pharmacogenetic test result. Treatment decisions should then be revised based on genotype results. Dual antiplatelet therapy is not recommended for secondary prevention in symptomatic LEAD.^23^
2. Chronic limb‐threatening ischaemia (CLTI).
  Pharmacogenetic testing for *CYP2C19* variants should be undertaken urgently. Most of these patients will already be on an antiplatelet agent at the time of presentation which should be continued, with the antiplatelet choice being amended based on the test result. For any CLTI patient not on an antiplatelet agent at the time of presentation, aspirin should be used until the genotyping result is available.
3. Post‐intervention.
  Intervention for symptomatic PAD and CLTI may be endovascular alone, open surgery alone or combined endovascular and open surgery (hybrid). Pharmacogenetic testing for *CYP2C19* variants should be requested in the pre‐operative period to guide post procedural prescription in combination with existing local policies and national guidelines on antiplatelet prescription after intervention.

Ischaemic events may also occur in other arterial circulations (for example, upper limb, renal or mesenteric). *CYP2C19* genotyping should be undertaken if clopidogrel is being considered for treatment.

### 5.4 Communication with primary care

In all the above scenarios, communication with primary care (and other clinical teams) is extremely important. This communication should provide the results of the *CYP2C19* pharmacogenetic testing, and whether clopidogrel prescribing needs to be initiated, modified or discontinued, based on the *CYP2C19* genetic test result. This decision—as well as recommendations for ongoing anti‐platelet treatment—should be made by the specialist clinical teams who have ordered the pharmacogenetic test for the patient prior to initiation, though sharing this information to support ongoing patient care is important and can be done by enabling the result to be entered in the patient's record (e.g., by highlighting relevant SNOMED CT codes to primary care practitioners, refer to Table 2). Primary care teams may also want to consult the position statement from the Royal College of General Practitioners (RCGP) on prescribing clopidogrel and the implication of genotyping (https://www.rcgp.org.uk/representing-you/policy-areas/genomic-position-statement).

**TABLE 2:** SNOMED‐CT codes for CYP2C19 metaboliser phenotypes.

SNOMED CT code wording	SNOMED CT code ID
Cytochrome P450 family 2 subfamily C member 19 poor metaboliser (finding)	738 786 005
Cytochrome P450 family 2 subfamily C member 19 intermediate metaboliser (finding)	738 787 001
Cytochrome P450 family 2 subfamily C member 19 normal metaboliser (finding)	738 788 006
Cytochrome P450 family 2 subfamily C member 19 rapid metaboliser (finding)	738 789 003
Cytochrome P450 family 2 subfamily C member 19 ultra‐rapid metaboliser (finding)	738 790 007

[Correction added on 26 December 2025, after first online publication: The SNOMED CT code ID in the fourth row has been corrected in this version.]

## 6 WHICH GENE(S), VARIANTS AND TURNAROUND TIME

The CYP2C19*1 (NG_008384.3:g.85186A > G) is the wild type allele and it is not normally tested. The following variants should be tested in *CYP2C19* when clopidogrel therapy is being initiated^24^:

Tier 1 variants – essential panel:

1. CYP2C19*2 (NG_008384.3:g.[17687A > G; 24179G > A; 85186A > G]) – loss‐of‐function allele
2. CYP2C19*3 (NG_008384.3:g.[22973G > A; 85186A > G]) – loss‐of‐function allele
3. CYP2C19*17 (NG_008384.3:g.[4220C > T; 85186A > G]) – gain‐of function allele

Tier 2 variants – extended panel:

1. CYP2C19*4 (NG_008384.3:g.[5026A > G; 85186A > G]) – loss of function allele
2. CYP2C19*5 (NG_008384.3:g.[85186A > G; 95058C > T])– loss of function allele
3. CYP2C19*6 (NG_008384.3:g.[17773G > A; 85186A > G]) – loss of function allele
4. CYP2C19*7 (NG_008384.3:g.[24 319 T > A; 85186A > G])– loss of function allele
5. CYP2C19*8 (NG_008384.3:g.[17 736 T > C; 85186A > G]) – loss of function allele
6. CYP2C19*9 (NG_008384.3:g.[17809G > A; 85186A > G]) – reduced function allele
7. CYP2C19*10 (NG_008384.3:g.[24178C > T; 85186A > G]) – reduced function allele
8. CYP2C19*35 (NG_008384.3:g.[17687A > G; 85186A > G]) – loss of function allele

[Correction added on 8 December 2025, after first online publication: The headings ‘Tier 1 variants – essential panel’ and ‘Tier 2 variants – extended panel’ have been added in this version.]

Variants highlighted in bold in the Tier 2 extended panel are likely to be relevant in the UK population^25^ based on the allele frequencies in different ethnic groups^2^ (Table 3) and therefore should be considered for inclusion in *CYP2C19* genotyping panels. It is important to note that implementation requirements will need to be updated with relevant changes in genotype‐to‐phenotype translations and allele definitions. This information can be obtained by the allele function assignments provided by the Clinical Pharmacogenetics Implementation Consortium (CPIC) in the Diplotype‐Phenotype Table available on the ClinPGx database.^2^

**TABLE 3:** Frequencies of *CYP2C19* alleles in different ethnic groups.

CYP2C19 allele	Sub‐Saharan African	African American/afro‐Caribbean	Asian (east)	Asian (central/south)	European	Latino	Oceanian
*1	0.55	0.55	0.60	0.54	0.63	0.72	0.19
*2	0.16	0.18	0.28	0.27	0.15	0.10	0.61
*3	0.003	0.003	0.07	0.02	0.002	0.001	0.15
*4	0.000	0.000	0.000	0.000	0.002	0.001	Not known
*5	0.000	0.000	0.003	0.000	0.000	0.000	Not known
*6	0.000	0.000	0.001	0.000	0.000	0.000	Not known
*7	0.000	0.000	0.000	0.000	0.000	0.000	Not known
*8	0.000	0.001	0.000	0.000	0.003	0.001	Not known
*9	0.03	0.014	0.000	Not known	0.001	0.001	Not known
*10	0.000	0.003	0.000	Not known	0.000	0.001	Not known
*17	0.17	0.21	0.02	0.17	0.22	0.17	0.06
*35	0.03	0.016	0.000	Not known	0.000	Not Known	Not known

Additional information on other CYP2C19 allelic variants can be found in the ‘CYP2C19 Frequency Table’ available through the ClinPGx database.^2^

As mentioned above, the variable combinations of these allelic variants can generate different phenotypes, whose frequency in different biogeographical groups is reported in Table 4.^2^

**TABLE 4:** Frequencies of *CYP2C19* phenotype in different ethnic groups (%).

CYP2C19 phenotype	Sub‐Saharan African	African American/afro‐Caribbean	Asian (east)	Asian (central/south)	European	Latino	Oceanian
Ultrarapid metabolizer	3.0	4.3	0	2.9	4.6	2.8	0.3
Rapid metabolizer	21.1	23.7	2.5	18.6	27.1	24.1	2.1
Normal metabolizer	37.0	32.8	38.1	29.6	39.6	52.5	3.5
Intermediate metabolizer	29.9	31.4	45.9	40.8	26.1	19.0	36.9
Poor metabolizer	3.7	4.1	13.0	8.2	2.4	1.1	57.1

Pharmacogenetic testing for *CYP2C19* genetic variants can be undertaken by laboratory testing or by point‐of‐care testing depending on availability, urgency of obtaining a genotype result and genotyping coverage of the different *CYP2C19* variants. For laboratory testing, the turnaround time within the laboratory should be 5 days or less.

Genotyping test results should be displayed in reports as per current best practice guidance alongside phenotype information (metaboliser type). This should then be recorded by the receiving clinician within the health record, ideally in the form of structured data, where this is available, for example, using SNOMED‐CT codes (Table 2). Therefore, laboratories may wish to highlight these codes in results.

## 7 CLINICAL ACTIONS BASED ON GENOTYPE

Prescribing recommendations should be guided by the pharmacogenetic test results as per Tables 5, 6, 7.

**TABLE 5:** Recommended clinical actions based on the pharmacogenetic test results in acute non cardioembolic ischaemic stroke or TIA.

Metaboliser phenotype	Prescribing suggestions	
	Minor ischaemic stroke or TIA	Non‐minor ischaemic stroke
Rapid and ultrarapid metaboliser	No pharmacogenetic informed action required. Follow the recommended prescribing guidelines taking account of the risk of bleeding and benefit into consideration	
Normal metaboliser	No pharmacogenetic informed action required. Clopidogrel can be prescribed at the standard dose (75 mg once daily).	
Intermediate metaboliser	Consider alternative antiplatelet agents/doses: Aspirin 75 mg once daily and Dipyridamole MR 200 mg twice daily OR Ticagrelor (180 mg loading dose followed by 90 mg twice daily) plus Aspirin (300 mg loading followed by 75 mg daily) for 30 days for high‐risk TIA and minor strokes (NIH Stroke Scale/Score [NIHSS] ≤ 3) eligible for DAPT, followed by Aspirin 75 mg once daily (+Dipyridamole MR 200 mg twice daily if tolerated) OR antiplatelet monotherapy with Ticagrelor 90 mg twice daily thereafter.	Consider alternative antiplatelet agents/doses: Aspirin 75 mg once daily plus Dipyridamole MR 200 mg twice daily
Poor metaboliser	Avoid Clopidogrel. Consider alternative antiplatelet agents/doses: Aspirin 75 mg once daily and Dipyridamole MR 200 mg twice daily OR Ticagrelor (180 mg loading dose followed by 90 mg twice daily) plus Aspirin (300 mg loading followed by 75 mg daily) for 30 days followed by Aspirin 75 mg once daily (+Dipyridamole MR 200 mg twice daily if tolerated) OR antiplatelet monotherapy with Ticagrelor 90 mg twice daily thereafter.	Avoid Clopidogrel. Consider alternative antiplatelet agents/doses: Aspirin 75 mg once daily plus Dipyridamole 200 mg MR twice daily

**TABLE 6:** Recommended clinical actions based on the pharmacogenetic test results in patients with CAD.

Metaboliser phenotype	Prescribing suggestions
Rapid and ultrarapid metaboliser	No pharmacogenetic informed action required. Follow the recommended prescribing guidelines taking account of the risk of bleeding and benefit into consideration.
Normal metaboliser	No pharmacogenetic informed action required. Clopidogrel can be prescribed at the standard dose (75 mg once daily).
Intermediate metaboliser	Consider alternative therapy with Aspirin (75 mg once daily) as DAPT with: Ticagrelor 90 mg twice daily, OR Prasugrel 10 mg once daily for the duration of time as recommended by standard international guidelines.
Poor metaboliser	Avoid Clopidogrel. Consider alternative therapy with Aspirin (75 mg once daily) as DAPT with: Ticagrelor 90 mg twice daily, OR Prasugrel 10 mg once daily for the duration of time as recommended by standard international guidelines. Where multiple medicines are available as alternatives, please consider suitability for the individual patient against local guidelines and formularies.

**TABLE 7:** Recommended clinical actions based on the pharmacogenetic test results in patients with LEAD.

Metaboliser phenotype	Prescribing suggestions
Rapid and ultrarapid metaboliser	No pharmacogenetic informed action required. Follow the recommended prescribing guidelines taking account of the risk of bleeding and benefit into consideration. Dual antiplatelet therapy may be needed after revascularisation procedures for 1–6 months.
Normal metaboliser	No pharmacogenetic informed action required. Clopidogrel can be prescribed at the standard dose (75 mg once daily). Dual antiplatelet therapy may be needed after revascularisation procedures for 1–6 months.
Intermediate metaboliser	Consider alternative antiplatelet agents/doses: Aspirin 75 mg once daily OR Aspirin 75 mg once daily + Rivaroxaban 2.5 mg twice daily
Poor metaboliser	Avoid Clopidogrel. Consider alternative antiplatelet agents: Aspirin 75 mg once daily OR Aspirin 75 mg once daily + Rivaroxaban 2.5 mg twice daily

### 7.1 Acute non‐cardioembolic ischaemic stroke or TIA

Where clopidogrel is considered to be the choice of therapy in patients with acute presentation of non‐cardioembolic stroke or TIA, the prescribing recommendations reported in Table 5 should be considered.

The following points need to be considered when prescribing for patients with acute non‐cardioembolic ischaemic stroke or TIA:

- Clinicians should also consult the relevant clinical guidelines from specialist societies,^5^ Royal Colleges, or NICE,^13^ when available as well as relevant local guidelines.
- Aspirin 75 mg once daily plus ticagrelor 90 mg twice daily may be used in the acute phase for up to 30 days. A loading dose of 300 mg once daily of aspirin is only required if >72 hrs since last dose of aspirin or naïve.
- There is limited safety data on the long‐term use of ticagrelor^26^, ^27^
- The use of prasugrel for stroke is contra‐indicated
- The licences for ticagrelor and clopidogrel include information on contraindications and cautions, and it is important that these are followed. Prescribers should also be aware of drug–drug interactions with ticagrelor and clopidogrel.
- Ticagrelor is recommended as an alternative anti‐platelet agent in the 2023 National Clinical Guideline for Stroke in the United Kingdom and Ireland^5^ although it is not licensed for use in stroke or TIA, and its use would be off label.
- Please see further discussion on the use of 150 mg once daily of clopidogrel in intermediate metabolisers below.
- In the absence of available relevant pharmacogenetic information or testing the current best practice clinical guidelines should be followed.

### 7.2 CAD

Where clopidogrel is considered to be the choice of therapy in patients with different clinical presentations of CAD, the prescribing recommendations reported in Table 6 should be considered.

The following points need to be considered when prescribing for patients with different presentations of CAD:

- Clinicians should also consult the relevant clinical guidelines from specialist societies,^28^, ^29^, ^30^, ^31^ Royal Colleges, or NICE, when available, as well as relevant local guidelines. Guidance should also be followed on the duration of antiplatelet therapy, including both monotherapy and dual therapy.
- Prasugrel has a licensed 5 mg dose for adults <60 kg and adults over 75 years.
- Loading doses of prasugrel (60 mg) or ticagrelor (180 mg) may need to be used in certain instances depending on clinical need. Local guidance should be followed for that.
- The licences for prasugrel, ticagrelor and clopidogrel include information on contraindications and cautions, and it is important that these are followed. Prescribers should also be aware of drug–drug interactions with ticagrelor, prasugrel and clopidogrel.
- If a patient is an intermediate or poor metaboliser, loading doses of clopidogrel should be avoided.
- Licensed doses of antiplatelet agents, or those recommended in clinical guidelines, and the recommended duration of therapy should be used whenever possible.
- In the absence of available relevant pharmacogenetic information or testing, the current best practice clinical guidelines should be followed.

### 7.3 PAD

For lower extremity arterial disease (LEAD), clopidogrel is considered to be the standard of care, but its use should be modified according to *CYP2C19* genotype as outlined in the Table 7.

The following points need to be considered when prescribing for patients with different presentations of PAD:

- The European Society for Vascular Surgery does not recommend dual antiplatelet therapy for secondary prevention in symptomatic LEAD.^23^
- Dual antiplatelet therapy may be needed after revascularisation procedures for a period of 1–6 months. This may be clopidogrel and aspirin (in ultra‐rapid and normal metabolisers). However, in intermediate and poor metabolisers, alternative regimens should be considered such as aspirin alone, or in those patients at high risk of ischaemic events but with low bleeding risk, the use of aspirin combined with low‐dose rivaroxaban, 2.5 mg twice daily. Please consult the relevant specialist guidelines.^20^, ^21^, ^23^, ^32^
- For CLTI, the time‐to‐treatment guidelines in the United Kingdom recommend revascularization within 5 days for hospitalized patients and 14 days for outpatients with CLTI. These timeframes are based on the UK's Vascular Society's Peripheral Arterial Disease Quality Improvement Framework (PAD‐QIF),^20^ emphasizing the urgency of treatment to prevent major amputation and mortality.
- Ticagrelor is not used in vascular surgery patients. It was found to be non‐superior to clopidogrel in the EUCLID^33^ study in preventing MACE events in patients with symptomatic PAD. Its use is not advocated in any of the above‐mentioned guidelines. Among the key exclusion criteria of this trial, there was the poor clopidogrel metaboliser status for the *CYP2C19* allele, defined as a genotype with two LOF alleles.
- For the treatment and prevention of atherothrombotic events in other arterial circulatory systems (upper limb, renal or mesenteric), if clopidogrel is used, please used the guidance in the table above. Please note that the evidence base on choice of antiplatelet agent(s) and duration of therapy is poor in these patients, and specialist guidelines should be consulted.^20^, ^21^, ^23^, ^32^
- The licences for clopidogrel and rivaroxaban include information on contraindications and cautions, and it is important that these are followed. Prescribers should also be aware of drug–drug interactions with ticagrelor and clopidogrel.
- In the absence of available relevant pharmacogenetic information or testing, the current best practice clinical guidelines should be followed

### 7.4 Use of alternatively dosed clopidogrel in CYP2C19 intermediate metabolisers

The use of alternatively dosed clopidogrel (150 mg once daily, 600 mg loading dose), which would be off label, has been suggested based on the patient's bleeding risk and drug efficacy considerations. The Dutch Pharmacogenetics Working Group (DPWG) guideline on clopidogrel, available through the ClinPGx database,^34^ recommends the use of 150 mg once daily of clopidogrel in CYP2C19 intermediate metabolisers, for those patients who have undergone percutaneous coronary intervention for whom the bleeding risk with prasugrel or ticagrelor is considered too high or for stroke/TIA patients who do not tolerate the combination of aspirin and dipyridamole. However, there is lack of consensus on its use since the CPIC^35^ and French National Network of Pharmacogenetics (RNPGx) guidelines^34^ do not recommend the use of 150 mg once daily of clopidogrel.

We have assessed the pharmacokinetic and clinical literature in this area and note that (a) the 150 mg dose of clopidogrel does lead to higher exposure to the active metabolite of clopidogrel than 75 mg; (b) the risk of bleeding is increased with 150 mg clopidogrel compared to 75 mg; and (c) the clinical benefit appears greater with ticagrelor than with 150 mg clopidogrel.

Therefore, we recommend the use of ticagrelor (when appropriate), or other non‐CYP2C19 metabolized antiplatelets if licensed, rather than 150 mg clopidogrel in intermediate metabolisers. We acknowledge, in certain patient populations, such as frail patients with multiple comorbidities, the bleeding risk associated with ticagrelor, or other antiplatelet agents may be considered too high. In such cases, clinical discretion is advised. If use of 150 mg once daily of clopidogrel is considered, the extent to which this reduces bleeding risk relative to alternatives remains uncertain, and careful clinical monitoring for bleeding is crucial.

## 8 OTHER PHARMACOGENETICS GUIDELINES

In this section, prescribing recommendations provided by other well established international pharmacogenetics consortia are summarized for comparison with the UK CERSI‐PGx guidelines.

### 8.1 The clinical pharmacogenetics implementation consortium guideline

CPIC published the first guideline on the use of *CYP2C19* genotyping to improve the prescription of clopidogrel in 2011,^36^ followed by two updates in 2013^37^ and 2022.^35^ The latest guideline^35^ recommends avoiding clopidogrel in CYP2C19 intermediate metabolisers (or likely intermediate metabolisers) and poor metabolisers (or likely poor metabolisers) and using an alternative antiplatelet agent, such as prasugrel or ticagrelor, if not contraindicated, in patients with acute coronary syndrome and/or undergoing PCI. The recommendations remain unchanged from previous guidelines. The strength of recommendations is ‘strong’ for both phenotypes, with a shift in the strength of recommendations from ‘moderate’ to ‘strong’ for CYP2C19 intermediate metabolisers. A standard dose of clopidogrel is recommended for CYP2C19 normal, rapid and ultrarapid metabolisers. These recommendations also apply to patients undergoing elective PCI. In contrast, there is a ‘moderate’ recommendation to avoid clopidogrel in CYP2C19 poor metabolisers (or likely poor metabolisers) in the other non‐ACS, non‐PCI cardiovascular indications. These include PAD and stable CAD following a recent myocardial infarction outside the PCI setting.

In the setting of neurovascular disease, the CPIC guideline recommends clopidogrel at standard doses in CYP2C19 normal metabolisers, with a ‘moderate’ recommendation to avoid clopidogrel in CYP2C19 poor metabolisers and in both intermediate and poor metabolisers to consider an alternative P2Y12 inhibitor at standard doses, in absence of contraindications. The guideline also highlights the haematological toxicity of ticlopidine (not licensed in the United Kingdom) and the contraindication of prasugrel in patients with a history of stroke and TIA.

The 2022 CPIC guideline does not support the use of 150 mg daily dose of clopidogrel in CYP2C19 intermediate metabolisers.^35^

### 8.2 The Dutch pharmacogenetics working group guideline

The DPWG has published therapeutic dose recommendations for clopidogrel based on *CYP2C19* genotype in 2011.^38^ The latest update on this gene‐drug pair was issued in 2018 and is available through the ClinPGx website.^34^ Currently, the DPWG recommends avoiding clopidogrel in CYP2C19 poor metabolisers undergoing percutaneous coronary intervention, or in those who have a stroke or TIA. The guideline recommends choosing an alternative drug, that is, ticagrelor or prasugrel after PCI, dipyridamole together with aspirin in stroke or TIA, or double the dose of clopidogrel to 150 mg once daily (with 600 mg loading dose) in CYP2C19 intermediate metabolisers. No action is required for CYP2C19 ultrarapid metabolisers. The DPWG underlines that ticagrelor and prasugrel are not metabolized by CYP2C19 or to a lesser extent than clopidogrel, respectively and are associated with an increased bleeding risk compared to clopidogrel. The DPWG has assigned a Clinical Implication Score of 8+ to *CYP2C19* pharmacogenetic testing in PCI and stroke/TIA patients prior to clopidogrel meaning that the test is ‘essential’ before starting therapy or shortly after initiation.

### 8.3 The French National Network of pharmacogenetics guideline

The RNPGx guideline for *CYP2C19* and clopidogrel is available via the ClinPGx database.^34^ The guideline recommends pharmacogenetic testing for the main *CYP2C19* deficiency alleles before clopidogrel administration. The pharmacogenetic test is considered essential for coronary angioplasty with stenting and potentially useful in other indications. The RNPGx recommends using an alternative treatment that is not a substrate of CYP2C19 (prasugrel, ticagrelor, acetylsalicylic acid), in subjects with at least one LOF allele. Based on the available evidence, the RNPGx does not recommend increasing the clopidogrel dose in subjects with one LOF allele. It also underlines that the CYP2C19*17 variant has been associated with increased risk of bleeding but also increased efficacy. There is not sufficient data to modify therapy in this subgroup of patients.

The ClinPGx database provides updated *CYP2C19* gene‐specific information tables, including new allelic variants, their function and frequency.^2^ In addition, the PGx Working Group of the Association for Molecular Pathology Clinical Practice Committee issues periodical recommendation on the *CYP2C19* variants that need to be tested as mentioned above.^24^

Table 8 provides a summary of current PGx guidelines.

**TABLE 8:** Summary of therapeutic recommendations based on different guidelines.

	CPIC			DPWG		RNPGx	UK CERSI‐PGx
CYP2C19 phenotype	Therapeutic recommendations ‐ classification of the recommendations			Therapeutic recommendations^f^		Therapeutic recommendations^j^	Therapeutic recommendations
	ACS and/or PCI	Non‐ACS, non‐PCI cardiovascular indications^b^ ^,^ ^c^	Neurovascular indications^d^	Percutaneous coronary intervention, stroke or TIA	Other indications	All indications	All licensed indications
Ultra‐rapid metaboliser	Standard dose (75 mg/day) ‐ Strong	No recommendation	No recommendation	No action required	No action required	No recommendation	No pharmacogenetic informed action required.
Rapid metaboliser	Standard dose (75 mg/day) ‐ Strong	No recommendation	No recommendation	Not considered in the guideline^**g**^	Not considered in the guideline^**g**^	No recommendation	No pharmacogenetic informed action required.
Normal metaboliser	Standard dose (75 mg/day) ‐ Strong	Standard dose (75 mg/day) ‐ Strong	Standard dose (75 mg/day) ‐ Strong	Not considered in the guideline	Not considered in the guideline	Not considered in the guideline	No pharmacogenetic informed action required.
Intermediate metaboliser	Avoid standard dose (75 mg) clopidogrel if possible^a^ – Strong	No recommendation	Consider an alternative P2Y12 inhibitor^e^ at standard dose if clinically indicated and no contraindication ‐ Moderate	Choose an alternative^h^ or double the dose to 150 mg/day (600 mg loading dose).	No action required	Alternative antiaggregant that is not a CYP2C19 substrate (prasugrel, ticagrelor, acetylsalicylic acid)^k^	Consider alternative drugs based on the indications.
Likely intermediate metabolizer^l^	Avoid standard dose clopidogrel (75 mg) if possible^a^ – Strong	No recommendation	Consider an alternative P2Y12 inhibitor^e^ at standard dose if clinically indicated and no contraindication ‐ Moderate	Not considered in the guideline	Not considered in the guideline	Not considered in the guideline	Consider alternative drugs based on the indications.
Poor metaboliser	Avoid clopidogrel if possible^a^ – Strong	Avoid clopidogrel if possible^a^ – Moderate	Avoid clopidogrel if possible. Consider an alternative P2Y12 inhibitor^e^ at standard dose if clinically indicated and no contraindication ‐ Moderate	Avoid clopidogrel^h^.	Determine the level of inhibition of platelet aggregation by clopidogrel. Consider an alternative in poor responders^i^.	Alternative antiaggregant that is not a CYP2C19 substrate (prasugrel, ticagrelor, acetylsalicylic acid)	Avoid clopidogrel and consider alternative drugs based on the indications
Likely poor metaboliser^l^	Avoid clopidogrel if possible^a^ – Strong	Avoid clopidogrel if possible^a^ – Moderate	Avoid clopidogrel if possible. Consider an alternative P2Y12 inhibitor^e^ at standard dose if clinically indicated and no contraindication ‐ Moderate	Not considered in the guideline	Not considered in the guideline	Not considered in the guideline	Avoid clopidogrel and consider alternative drugs based on the indications.

*Note*: Consider that exact wording from the guidelines is reported in the table. For more information on the DPWG and RNPGx guidelines refer to the ClinPGx website.^34^

^a^ Use prasugrel or ticagrelor at standard dose if no contraindication.

^b^ Non‐ACS, non‐PCI cardiovascular indications include PAD and stable CAD following a recent myocardial infarction outside the setting of PCI.

^c^ Prasugrel and ticagrelor are not approved for elective PCI by the FDA and MHRA.

^d^ Ticagrelor is currently FDA approved to reduce the risk of stroke in patients with acute ischemic stroke (NIH Stroke Scale score ≤5) or high‐risk transient ischemic attack (TIA).

^e^ Alternative P2Y12 inhibitors not impacted by *CYP2C19* genetic variants include ticagrelor and ticlopidine. Prasugrel is contraindicated in patients with a history of stroke or TIA. Given limited outcomes data for genotype‐guided anti‐platelet therapy for neurovascular indications, selection of therapy should depend on individual patient treatment goals and risks for adverse events.

^f^ The Dutch Pharmacogenetics Working Group (DPWG) considers genotyping before starting clopidogrel in PCI or stroke patients to be essential for drug efficacy. Genotyping must be performed before drug therapy has been initiated to guide drug and dose selection.

^g^ The DPWG do not report rapid metaboliser since this has been grouped with the normal metaboliser phenotype.

^h^ Prasugrel, ticagrelor and acetylsalicylic acid/dipyridamole are not metabolized by CYP2C19 (or to a lesser extent).

^i^ Prasugrel and ticagrelor are not metabolized by CYP2C19 (or to a lesser extent).

^j^ Testing for the main *CYP2C19* deficiency alleles before instituting clopidogrel treatment is recommended (the test is essential for coronary angioplasty with stenting and based on the current state of knowledge this test is potentially useful in the other indications).

^k^ Based on current knowledge, it is not recommended to increase the clopidogrel dose in patients with the CYP2C19*2 or *3 allele.

^l^ The likely intermediate metaboliser and likely poor metaboliser phenotypes can occur if an extended panel of *CYP2C19* variants is used with variants that have not been assigned a reduced activity, like CYP2C19*9.^35^ In these cases, the CERSI‐PGx recommendations applied to IMs and PMs should be considered.

## 9 HEALTH ECONOMIC EVALUATION

### 9.1 Stroke and transient ischaemic attack

NICE assessed two point‐of‐care genotype tests (Genedrive CYP2C19 ID Kit and Genomadix Cube CYP2C19 system) and laboratory testing to guide clopidogrel use after ischaemic stroke or transient ischaemic attack.^13^ The cost per test was calculated as £104 for Genedrive, £197 for Genomadix Cube and and £139 for laboratory testing (reducing to £44 with testing in batches of 55). The economic model informing the assessment was based on a decision tree used to capture short‐term (90 day) outcomes, and a Markov model with a 1‐year cycle to capture outcomes over a patient's life‐time.^12^ Testing done for people with non‐minor stroke resulted in positive incremental net monetary benefit (INMB) irrespective of the method used. While still positive, the INMB was lower for those with minor stroke or transient ischaemic attack, with values ranging from £753 to £1131. Tests remained cost effective when the choice of antiplatelet and timing of treatment were varied. Compared with no testing, the cost of each test would have to be much higher (around £1900 higher for Genedrive, £1800 higher for the Genomadix Cube and £1920 for laboratory‐based testing) for testing not to be cost effective.

The main limitation of the analysis was the absence of studies to provide direct evidence on the efficacy of genotype testing. As a result, the economic model relied on indirect evidence to estimate the effects of alternative antiplatelet therapies depending on LOF status.^39^

Although there was little difference in the QALYs generated by the different methods of testing, NICE stated a preference for laboratory‐based tests over point‐of‐care tests because of the greater flexibility available in relation to testing for new variants, the likelihood of introducing greater health inequalities with point‐of‐care tests left to local decision makers, the difficulty in ensuring standardization in quality assurance, and the likely future shift from reactive to pre‐emptive genotyping.^13^ However, the committee did note that the estimated cost per test for Genedrive is less than for Genomadix Cube and concluded that Genedrive was its preferred point‐of‐care test. This was also based on other advantages of the Genedrive platform including the fact that reagents do not need to be stored in a freezer and the flexibility to detect additional alleles including those that occur at a greater frequency in some ethnic groups.

### 9.2 CAD

The economic evidence for single gene (*CYP2C19*) pharmacogenetic testing relates to patients with acute coronary syndrome.^40^, ^41^, ^42^, ^43^ Of 24 published analyses, 19 examined the effect of escalating antiplatelet treatment from clopidogrel to ticagrelor or prasugrel in patients found to have a loss‐of‐function allele. The majority of cost utility analyses (14/21) considered costs from the perspective of payers in the USA, with the remaining coming from a wide variety of jurisdictions among European, Middle eastern and Asian countries including Hong Kong, Singapore and China. There were no UK economic evaluations. In nine analyses, a genotype guided strategy was found to dominate all other options; among the remaining economic analyses, universal ticagrelor was found to be most cost effective in 3, a genotype approach most cost effective in 8 and a non‐guided de‐escalation approach in 1. While the economic evidence lends support to *CYP2C19* pharmacogenetic testing in patients with acute coronary syndrome, cost‐effective analyses that are relevant to the NHS in the United Kingdom are required.

### 9.3 PAD

No economic evaluations were identified for *CYP2C19* pharmacogenetic testing in relation to the use of clopidogrel in PAD.

## 10 REGULATORY CONSIDERATIONS

### 10.1 Summary of product characteristic (SmPC)^4^

The UK SmPC highlights the role of *CYP2C19* genetic polymorphisms in clopidogrel metabolism and platelet function in Section 4.4 of the document. It also describes some of the evidence of this genetic polymorphism on clinical outcomes (in Section 5.2 of the SmPC), but states that ‘The influence of *CYP2C19* genotype on clinical outcomes in patients treated with clopidogrel has not been evaluated in prospective, randomised, controlled trials’, indicating that it has not been updated with the latest evidence. The SmPC does not recommend pharmacogenetic testing for *CYP2C19* prior to clopidogrel therapy, but states that tests are available. The SmPC however does recommend the avoidance of concomitantly administered drugs that are known to be strong or moderate CYP2C19 inhibitors, since they can reduce the formation of the active metabolite of clopidogrel. Clinical actions based on genotype recommended by this guideline are not included in Section 4.2 of the SmPC of clopidogrel containing medicinal products.

Ticagrelor is not licensed for the treatment of stroke/TIA in the United Kingdom or EU. In 2021, the licence holder withdrew its application to the European Medicines Agency (EMA) for use in stroke/TIA. At the time of withdrawal, the EMA was of the opinion that the benefits did not outweigh the risks. However, ticagrelor is authorized by the FDA ‘to reduce the risk of stroke in patients with acute ischemic stroke (NIH Stroke Scale score ≤5) or high‐risk transient ischemic attack (TIA)’. As mentioned previously, ticagrelor is recommended as an alternative anti‐platelet agent in the 2023 National Clinical Guideline for Stroke in the United Kingdom and Ireland.^5^

### 10.2 NICE diagnostics guidance (DG59 – published 31 July 2024)^13^

NICE has recommended the use of *CYP2C19* pharmacogenetic testing to determine if clopidogrel is a suitable antiplatelet agent for people who have just had an ischaemic stroke or a transient ischaemic attack. NICE recommends the use of lab‐based testing, or point‐of‐care testing when lab‐based testing is not available. Retrospective pharmacogenetic testing of people already on clopidogrel was considered to be outside the scope of assessment and the guidance noted that the risk of recurrent stroke or TIA is highest in the first 90 days after stroke or TIA and then decreases. Pharmacogenetic testing of people with CAD and other indications for the use of clopidogrel was also outside the scope of assessment.

## 11 OTHER CONSIDERATIONS

- This guideline focuses on *CYP2C19* genotyping in people being considered for clopidogrel. Variants in other genes (*CES1*, *ABCB1* and *P2Y12*) have been investigated as determinants for the effectiveness of clopidogrel, but the data have been contradictory and thus are outside the scope of this guideline.
- Phenoconversion occurs when genotypic extensive metabolisers are converted into phenotypic poor metabolisers by the use of drugs which inhibit CYP2C19 while they are also on clopidogrel. The UK SmPC advises against the use of strong or moderate CYP2C19 inhibitors in patients on clopidogrel (e.g., omeprazole and fluoxetine). Conversely, increased activity of the CYP2C19 enzyme can occur with the concomitant administration of enzyme inducers including but not limited to rifampin, phenytoin, carbamazepine and St John's wort.
- CYP2C19 is also involved in the metabolism of other drugs such as antidepressants (SSRIs and TCA) and voriconazole.
- *CYP2C19* genotyping is currently performed at a national level for the prescription of mavacamten, a new myosin inhibitor approved by the MHRA for the treatment of obstructive hypertrophic cardiomyopathy.^44^

## 12 RESEARCH RECOMMENDATIONS

During the development of these guidelines, it became evident that several areas lack sufficient supporting evidence. In the following section, we outline research recommendations intended to highlight key opportunities for further investigation. While not exhaustive, these suggestions offer insight into where additional research could strengthen the evidence base for the use of clopidogrel in atherosclerotic arterial disease.

### 12.1 Variation in CYP2C19 activity

- There are many *CYP2C219* allelic variants where the functional effects are unclear. Thus, further work to assess their functional effects would help in improving the phenotype–genotype relationship of CYP2C19.
- CYP2C19*17 is associated with increased activity of the CYP2C19 enzyme. Theoretically, this could increase the risk of bleeding associated with clopidogrel. However, the evidence for this is contradictory, and further work is needed to determine whether having the CYP2C19*17 allele increases the risk of bleeding overall, and in particular, in certain patient sub‐groups, such as the elderly.
- Further work is needed to better understand the utility of 150 mg of clopidogrel in CYP2C19 intermediate metabolisers in terms of clopidogrel pharmacokinetics and clinical efficacy and safety.
- The issue of phenoconversion with concomitant drugs has been mentioned in this guideline. However, there is also evidence that severe forms of non‐alcoholic steatohepatitis reduce the expression of *CYP2C19* at the mRNA level, and potentially activity. Further work is required to determine (a) whether clopidogrel use provides inadequate protection in these patients (irrespective of genotype), and (b) whether the same effect is also seen in other forms of severe liver disease where inflammation is prominent.
- There is a need for further research on the utility of *CYP2C19* genotyping in PAD^45^ (lower limb, upper limb, renal, etc.), including health economic evaluation.
- Health economic analysis of *CYP2C19* genotyping in CAD in the UK setting is needed.

### 12.2 Efficacy and safety of antiplatelet agents

- There are limited safety data on the long‐term use of ticagrelor in stroke, CAD and PAD. This is an area that deserves further investigation. Considering the likely wide implementation of *CYP2C19* pharmacogenetic testing in this therapeutic area, follow up of patients prescribed ticagrelor according to this guideline may fill in the gap.
- Ticagrelor is not currently recommended for major stroke, and the need for a randomized controlled trial assessing the efficacy and safety needs to be considered.
- The clinical effectiveness of genotyping and antiplatelet switching in prevalent clopidogrel users (i.e., use greater than 6 months since the acute indication) in the three main clinical areas needs to be undertaken, together with an omnibus analysis.

### 12.3 Platelet function tests (PFTs)

The role of platelet function testing in assessing the response to clopidogrel has not been fully validated. The 2024 update of the International Consensus Statement on Platelet Function and Genetic Testing in Percutaneous Coronary Intervention^46^ provides different cut‐off points for different PFTs to define the high and low platelet reactivity. The Consensus Statement also highlights the limitations of platelet function assays, including the uncertainty on the optimal timing of testing with relation to the PCI, inter‐assay and intra‐patient variability of readouts, difficulties in using these tests in the context of ACS and during de‐escalation from prasugrel/ticagrelor to clopidogrel. Therefore, there is a need for further research on the role PFTs can play in determining the optimal use of clopidogrel and other antiplatelet agents, whether they should be used instead of pharmacogenetic testing or in conjunction with pharmacogenetic testing, especially in intermediate metabolisers.

## CONFLICT OF INTEREST STATEMENT

The conflict of interest statements are documented in Table S1.

**BOX 1 Summary guidance for *CYP2C19* pharmacogenetic testing when using clopidogrel in cerebrovascular disease (developed by the UK CERSI‐PGx).**

**RECOMMENDED INDICATIONS for PHARMACOGENETIC TESTING.**

Any patient who is about to be prescribed clopidogrel, regardless of the underlying indication, should undergo pharmacogenetic testing to identify clinically relevant *CYP2C19* polymorphisms, where testing is available, to optimize their antiplatelet therapy.

**INTEGRATING PHARMACOGENETIC TESTING INTO EXISTING CLINICAL PATHWAYS.**

***Transient ischaemic attack and minor stroke***: Pharmacogenetic testing for *CYP2C19* should be done when a diagnosis of TIA or minor ischemic stroke is made.

***Non‐minor ischaemic stroke***: Pharmacogenetic testing for *CYP2C19* should be done at the time of stroke after haemorrhage has been excluded by CT scanning and in the absence of an immediate cardioembolic cause.

In the absence of available relevant pharmacogenetic information or testing the current best practice clinical guidelines should be followed.

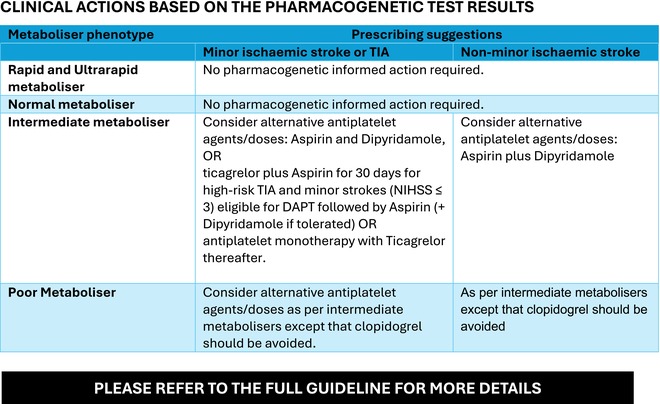

**BOX 2 Summary guidance for *CYP2C19* pharmacogenetic testing when using clopidogrel in coronary artery disease (developed by the UK CERSI‐PGx).**

**RECOMMENDED INDICATIONS for PHARMACOGENETIC TESTING.**

Any patient who is about to be prescribed clopidogrel, regardless of the underlying indication, should undergo pharmacogenetic testing to identify clinically relevant *CYP2C19* polymorphisms, where testing is available, to optimize their antiplatelet therapy.

**INTEGRATING PHARMACOGENETIC TESTING INTO EXISTING CLINICAL PATHWAYS.**

Testing for *CYP2C19* genetic variants in people with CAD should take into the account: (a) acuity of clinical presentation, (b) concomitant use of oral anticoagulants (OACs), (c) risk of bleeding and recurrent CVD events in the individuals treated with clopidogrel. In the context of acute coronary syndromes, clopidogrel pharmacogenetic testing is recommended at the time of hospital admission for those individuals in whom clopidogrel is deemed to be the more suitable alternative to other potent P2Y12 inhibitors (due to high bleeding risk or concomitant use of OACs). In individuals diagnosed with chronic coronary syndromes (CCS, previously known as ‘stable CAD’), clopidogrel testing is recommended upon first contact with a specialist centre involved in either outpatient or day‐case management of these individuals (e.g., individuals attending cardiac catheterisation procedures). Regardless of the clinical context, initiation of clopidogrel (if deemed appropriate) should not be delayed following the first contact but must be modified based on the results of the pharmacogenetic test.

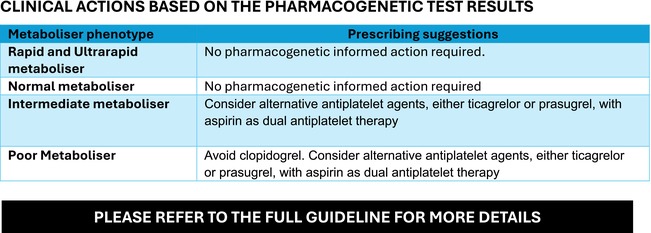

**BOX 3. Summary guidance for *CYP2C19* pharmacogenetic testing when using clopidogrel in peripheral arterial disease (developed by the UK CERSI‐PGx)**

**RECOMMENDED INDICATIONS for PHARMACOGENETIC TESTING.**

Any patient who is about to be prescribed clopidogrel, regardless of the underlying indication, should undergo pharmacogenetic testing to identify clinically relevant *CYP2C19* polymorphisms, where testing is available, to optimize their antiplatelet therapy.

**INTEGRATING PHARMACOGENETIC TESTING INTO EXISTING CLINICAL PATHWAYS.**

For lower limb ischaemia, there are three broad clinical scenarios:

1. For symptomatic lower extremity arterial disease (e.g., intermittent claudication), aspirin should be commenced while waiting for the *CYP2C19* pharmacogenetic test result. Treatment decisions could then be revised based on genotype results.
2. For chronic limb‐threatening ischaemia, *CYP2C19* pharmacogenetic testing should be undertaken urgently. Current antiplatelet treatment should be amended based on the genotype test result.
3. In patients due to undergo surgical or endovascular revascularisation, pharmacogenetic testing for *CYP2C19* variants should be requested in the pre‐operative period to guide post‐procedural prescription. Post‐procedural antiplatelet regimen is guided by assessment of genotype status, bleeding risk and type of revascularisation performed.

Ischaemic events may also occur in other arterial circulations (for example, upper limb, renal or mesenteric). *CYP2C19* genotyping should be undertaken if clopidogrel is being considered for treatment.

In the absence of available relevant pharmacogenetic information or testing the current best practice clinical guidelines should be followed.

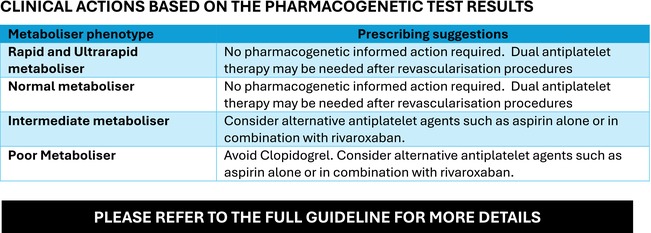

## Supporting information


**Table S1.** Competence, affiliations, and disclosure of conflicts of interest of the writing committee of the CERSI‐PGx Guideline for CYP2C19 Genotyping with Clopidogrel.
**Table S2.** Comments received during the Consultation period by various organizations on the CERSI‐PGx Guideline for CYP2C19 Genotyping with Clopidogrel and Responses by the UK CERSI PGx writing committee.

## Data Availability

The data that support the findings of this guideline are publicly available, as referenced.
