# Supplementary material for: CYP2C19 genotype testing for clopidogrel: A guideline developed by the UK Centre of Excellence in Regulatory Science and Innovation in Pharmacogenomics (CERSI‐PGx)
Source: Br J Clin Pharmacol. 2025 Dec 3;92(2):329–47. doi: 10.1002/bcp.70370 (PMC12850553; doi:10.1002/bcp.70370)
Supplement: Supplementary file 1 — Table S1. Competence, affiliations, and disclosure of conflicts of interest of the writing committee of the CERSI‐PGx Guideline for CYP2C19 Genotyping with Clopidogrel. Table S2. Comments received during the Consultation period by various organizations on the CERSI‐PGx Guideline for CYP2C19 Genotyping with Clopidogrel and Responses by the UK CERSI PGx writing committee. [file BCP-92-329-s001.docx]

***CYP2C19* genotype testing for clopidogrel: a guideline developed by the UK Centre of Excellence for Regulatory Science and Innovation in Pharmacogenomics (CERSI-PGx)**

Cinzia Dello Russo^1,2^, Iain Frater^3^, Rebecca Kuruvilla^1,4^, Stefanie Lip^3,5^, Hannah O’Neill^6^, Kerry Burke^7,8,9^, Vicky Chaplin^10^, Alexander SF Doney^11^, Salim Elyas^12,13^, Nicholas Graves^7^, Sophie Harding^14^, David Hargroves^15^, Judith Hayward^16^, Dyfrig A Hughes^17^, Tom AT Hughes^18^, Sree Kondapally^19,20^, Patrick Mok^21^, Aaron Peace^22^, Imran Rafi^23^, Simon Ray^24^, Vicky Stinton^25^, Luigi Venetucci^26^, Munir Pirmohamed^1,4,*^.

^1^Department of Pharmacology and Therapeutics, Institute of Systems, Molecular and Integrative Biology, University of Liverpool, Liverpool, UK.

^2^Department of Healthcare Surveillance and Bioethics, Section of Pharmacology, Università Cattolica del Sacro Cuore - Fondazione Policlinico Universitario A. Gemelli, IRCCS, Rome, Italy.

^3^School of Cardiovascular and Metabolic Health, University of Glasgow, Glasgow, UK.

^4^The Wolfson Centre for Personalised Medicine, Centre for Drug Safety Science, University of Liverpool, Liverpool, UK.

^5^Queen Elizabeth University Hospital, Glasgow, UK.

^6^East of Scotland Vascular Network, Department of Vascular Surgery, University of Dundee, UK

^7^Department of Vascular Surgery, Manchester Royal Infirmary, Manchester University NHS Foundation Trust, Manchester, UK

^8^Manchester Centre for Genomic Medicine, St Mary’s Hospital, Manchester University NHS Foundation Trust, Manchester, UK;

^9^Division of Evolution, Infection and Genomics, School of Biological Sciences, The University of Manchester, UK

^10^Genomics Unit, NHS England, UK

^11^School of Medicine, University of Dundee, Dundee, UK.

^12^The Academic Department of Healthcare for Older People, Royal Devon University Healthcare NHS Foundation Trust, Exeter, UK.

^13^Department of Clinical and Biomedical Science, University of Exeter Medical School, Exeter, UK.

^14^Velindre Cancer Centre, Cardiff and Vale University, Cardiff, UK

^15^Department of Stroke Medicine, East Kent Hospitals University NHS Foundation Trust, Canterbury, Kent, UK.

^16^NW GMSA, NHSE Genomics Education Programme, Affinity Care, UK.

^17^Centre for Health Economics and Medicines Evaluation, North Wales Medical School, Bangor University, Bangor, UK

^18^Neurology, University Hospital of Wales Healthcare NHS Trust, Cardiff, UK.

^19^Cardiovascular Clinical Academic Group, Molecular and Clinical Sciences Research Institute, St. George's University of London, London, UK

^20^St. George's University Hospitals NHS Foundation Trust, London, UK

^21^Doncaster and Bassetlaw Teaching Hospitals NHS Foundation Trust, Doncaster, UK

^22^Western Health and Social Care Trust, Department of Cardiology and Clinical Translational Research and Innovation Centre, Ulster University, Londonderry, UK.

^23^St. George's University of London, London, UK.

^24^Department of Cardiology, Manchester University NHS Foundation Trust, Manchester, UK.

^25^North West GLH, The University of Manchester, Manchester, UK

^26^Faculty of Biology, Medicine and Health, The University of Manchester, Manchester, UK

**Supplementary Methodology**

**Writing committee**

The UK CERSI-PGx guideline on *CYP2C19* genotype testing for clopidogrel was developed by a multidisciplinary team, including experts in clinical pharmacology, pharmacology and pharmacogenomics, clinicians, including neurologist and stroke physicians, cardiologists, a vascular surgeon, and two general practitioners; experts in pharmacy, genomic medicine and health economics. The guideline committee included three members from Scotland, three from Wales and one from Northern Ireland in addition to members from England. Full disclosure of the affiliations, competences and conflict of interests is provided in a separate document in the supplement. The writing committee convened the first time online on the 19 of February 2025 and subsequently met online four times (on 14 March, 11 April, 13 May and 24 June) to finalize the guideline draft for consultation, with written contributions from members throughout this period. Details on the writing committee are provided in Supplementary Table 1.

**Standardized template**

The first step undertaken by this committee was the development of a standardized template for the UK CERSI-PGx guidelines. A draft template was presented to the committee members on the first meeting and wording on the template modified as the guideline developed. It was also shared with regulators at the Medicines & Healthcare products Regulatory Agency (MHRA).

**Literature review and prescribing recommendations**

Recommendations from the UK CERSI-PGx consortium are evidence-based together with expert input from committee members who were able to highlight nuances in specific clinical settings in which clopidogrel is used. A comprehensive literature review was conducted on the three main areas of clinical use of clopidogrel, integrating information provided by the latest CPIC guideline published in 2022^1^ and recommendations from the DPWG, available through the ClinPGx database^2^. We also consulted other guidelines/guidance produced by specialist societies and by NICE (a few members of our committee had also served on the NICE committee which produced the NICE Diagnostics guidance DG59 on CYP2C19 genotyping after transient ischaemic attack and ischaemic stroke^3^). In the Evidence Overview section, a summary of the most relevant evidence is provided. We included data from recent randomised clinical trials and meta-analyses. An extensive literature review was performed on specific topics, including the use of alternatively dosed clopidogrel in cardiovascular and cerebrovascular disease management, and in relation to the cost-effectiveness of genotyping, prioritizing studies relevant to the UK NHS when available. The section on Clinical actions based on genotype contains prescribing recommendations for alternative treatments that are based on the alternative drugs available in the UK and on what is the UK routine practice as suggested by the clinical members of the writing committee. Whenever possible relevant UK prescribing guidelines have been quoted.

**Genetic variants to be tested**

The selection of *CYP2C19* genetic variants of to be tested was based on the recommendations of the American Association for Molecular Pathology, comprising both Tier 1 variants (minimum panel) and Tier 2 variants (extended panel)^4^. The allele function assignments provided by the CPIC and the diplotype-to-phenotype tables available through the ClinPGx database are indicated as reliable and updated sources for genotype-to-phenotype translations and allele definitions.

**Consultation process**

At the end of the writing process, the guideline final draft was shared in confidence for consultation with the Regulators (the MHRA, The National Institute for Health and Care Excellence), Specialty Societies relevant to the prescription of clopidogrel, Pharmacy Professional Networks, the CPIC and the DPWG, Genomic Societies, like the Association for Molecular Pathology and Association for Clinical Genomic Science, the Association of the British Pharmaceutical Industry, The British in vitro Diagnostics Association, and the UK-PGx Industry Network. The consultation period started on the 15 of July 2025 and was open for one month, with a month extension to collect input from most stakeholders. Comments received are included in a single table together with the UK CERSI-PGx responses to each of the comments, and if appropriate, by changes in the guideline. The content of the table is published as Supplementary Table 2 to ensure transparency of the procedures undertaken.

**Patient And Public Involvement and Engagement (PPIE)**

The clopidogrel guideline was shared with two patients’ representatives, members of the PPIE group established for the delivery of work package 5 of the UK CERSI-PGx project^5^. Patients participated in the last on-line meeting held in June 2025. A lay summary has been prepared with their contribution to explain the relevance of genetic variability on the clopidogrel benefits/risks profile and the impact of genotyping on its prescription. The lay summary is included in the supplementary material, and also on the UK CERSI-PGx website (in development).

**Supplementary References**

1. Lee CR, Luzum JA, Sangkuhl K, et al. Clinical Pharmacogenetics Implementation Consortium Guideline for CYP2C19 Genotype and Clopidogrel Therapy: 2022 Update. Clin Pharmacol Ther. 2022;112(5): 959-967. doi: 10.1002/cpt.2526.
2. The ClinPGx database - <https://www.clinpgx.org/chemical/PA449053/prescribingInfo> [Accessed on 07 October 2025].
3. National Institute for Health and Care Excellence. CYP2C19 genotype testing to guide clopidogrel use after ischaemic stroke or transient ischaemic attack. DG59. July 2024. <https://www.nice.org.uk/guidance/dg59>
4. Pratt VM, Del Tredici AL, Hachad H, et al. Recommendations for Clinical CYP2C19 Genotyping Allele Selection: A Report of the Association for Molecular Pathology. *J Mol Diagn*. 2018; 20(3): 269-276. doi: 10.1016/j.jmoldx.2018.01.011
5. Pirmohamed M and Dello Russo C. UK Centre of Excellence in Regulatory Science and Innovation in Pharmacogenomics. *Br J Clin Pharmacol*. 2025. *In press.*

**Supplementary Plain English Summary**

Kamil Sterniczuk, member of the UK CERSI-PGx PPIE group.

**What is clopidogrel?**

Clopidogrel is a medicine used to stop dangerous blood clots. Doctors usually prescribe it after a heart attack, stroke or when a patient has a stent fitted in their heart arteries. It works by making blood less likely to clot, which reduces the chance of another serious event. It is also given when peripheral arteries are narrowed by atherosclerosis to prevent occlusion by clots.

**Why does CYP2C19 matter?**

For clopidogrel to work, it must be changed into its active form by the liver. This process depends on a gene called *CYP2C19*. People inherit different versions of this gene, which means the medicine works better for some than for others. If the gene works normally, clopidogrel should be effective. If it works less well, the drug may not protect against clots, leaving patients at higher risk.

**Different types of response**

Some people are known as poor metabolisers because their bodies cannot convert enough clopidogrel into its active form. Others are intermediate metabolisers, where the effect is reduced but not completely lost. Normal metabolisers process the medicine as expected, while rapid and ultra-rapid metabolisers convert it more quickly but usually without problems.

**What do the guidelines say?**

The guideline recommends that doctors consider a genetic test to check how well a patient can process clopidogrel. This is especially useful after mini-strokes or strokes or when the risk of clots is high. If a patient is found to be a poor or intermediate metaboliser, the advice is to use another medicine, such as ticagrelor, which does not rely on the *CYP2C19* gene. If the patient is a normal or rapid/ultra-rapid metaboliser, clopidogrel can be prescribed with confidence. In urgent situations, doctors may begin clopidogrel treatment straight away so there is no delay. When test results are available, they can decide whether to continue or switch to a different drug.

**What this means for patients**

Testing helps doctors make sure each patient receives the drug that will work best for them. The results should be recorded in medical records so that all healthcare teams know the safest and most effective treatment plan. Patients should also be told why the test is important and what the results mean for their care.

**Health economic considerations**

Testing adds costs but may be cost-effective in the long run by preventing strokes, heart attacks, and blocked stents or blocked peripheral arteries, which are expensive to treat. The guideline highlights the importance of further economic evaluation in real-world NHS settings.

**Regulatory considerations**

Different countries may vary in how they approve and support genetic testing. The guideline notes that regulatory bodies should ensure tests are accurate, affordable, and available quickly enough to influence urgent treatment decisions.

**Research recommendations**

More evidence is needed on:

- The cost-effectiveness of genetic testing particularly in coronary artery disease and peripheral vascular disease.
- The impact of testing across diverse patient groups.
- How best to integrate genetic testing into everyday care without delaying treatment.
- The benefits of routine genetic testing for CYP2C19 in patients on clopidogrel to the NHS over the longer term.

**Key message**

Clopidogrel is an important drug for preventing blood clots but it does not work well for everyone. A simple genetic test can show whether it is the right medicine. If not, safe and effective alternatives are available. This approach makes treatment more personal, giving patients better protection against heart attacks and strokes.

**Supplementary Table 1.** *Competence, affiliations, and disclosure of conflicts of interest of the writing committee of the CERSI-PGx Guideline for CYP2C19 Genotyping with Clopidogrel.*

| **First Name Surname** | **Main Expertise** | **Full Affiliation** | **Institution/s** | **Conflict of Interest** | **Nation** |
| --- | --- | --- | --- | --- | --- |
| **Munir Pirmohamed** | Clinical Pharmacology Internal Medicine  Pharmacogenomics | Department of Pharmacology and Therapeutics, Wolfson Centre for Personalised Medicine, University of Liverpool, Liverpool, UK.  Liverpool University Hospital Foundation NHS Trust, Liverpool, UK. | University of Liverpool | Currently receives partnership funding, paid to the University of Liverpool, for the MRC Medicines Development Fellowship Scheme (co-funded by MRC and GSK, AZ, Optum and Hammersmith Medicines Research). He has developed an HLA genotyping panel with MC Diagnostics but does not benefit financially from this. He is part of the IMI Consortium ARDAT ([www.ardat.org](http://www.ardat.org)); none of these of funding sources have been used for the guideline. | England |
| **Cinzia**  **Dello Russo** | Pharmacology  Pharmacogenomics | Department of Pharmacology and Therapeutics, Institute of Systems Molecular and Integrative Biology (ISMIB), University of Liverpool, Liverpool, UK.  Department of Healthcare Surveillance and Bioethics, Section of Pharmacology, Università Cattolica del Sacro Cuore, Fondazione Policlinico Universitario A. Gemelli IRCCS, Rome, Italy, | University of Liverpool  Università Cattolica del Sacro Cuore | No conflicts | England |
| **Aaron Peace** | Cardiology | Western Health and Social Care Trust, Department of Cardiology and Clinical Translational Research and Innovation Centre. Ulster University, Northern Ireland, United Kingdom. | Western Health and Social Care Trust, Ulster University. | No conflicts | Northern Ireland |
| **Alexander SF Doney** | Stroke Medicine  Clinical Pharmacology  Internal Medicine  Pharmacogenomics | School of Medicine, University of Dundee, Dundee, UK. | University of Dundee | Led on implementing clopidogrel pharmacogenomics in stroke in NHS Tayside | Scotland |
| **Dyfrig A Hughes** | Health Economics  Pharmacy  Pharmacogenomics | Centre for Health Economics and Medicines Evaluation, North Wales Medical School, Bangor University, Bangor, United Kingdom | Bangor University | Chair of NHS Wales National Pharmacogenomics Group, and co-chair of NHS England Pharmacogenomics Test Evaluation Working Group | Wales |
| **Hannah O’Neill** | Vascular Surgery | East of Scotland Vascular Network, Department of Vascular Surgery | University of Dundee | No conflicts | Scotland |
| **Iain Frater** | Clinical pharmacology Internal Medicine | School of Cardiovascular and Metabolic Health, University of Glasgow, Glasgow, Scotland, UK | University of Glasgow | No conflicts | Scotland |
| **Imran Rafi** | Primary Care  Genomics | St. George's University of London, London. | St George’s, University of London | RCGP Joint Clinical Representative in Genomic Medicine with Dr. Judith Hayward | England |
| **Judith Hayward** | Primary Care  Genomics | NW GMSA, NHSE Genomics Education Programme, Affinity Care. | NW GMSA, NHSE Genomics Education Programme, Affinity Care. | GPwER in Clinical Genetics/Genomics, Leeds Clinical Genomics Service; Innovation Director, NW GMSA; Primary Care Lead, National Genomics Education Programme; RCGP Joint Clinical Representative in Genomic Medicine with Dr. Imran Rafi; Honorary Research Fellow, City St. George's University London; GP, Shipley Medical Practice, Affinity Care | England |
| **Luigi Venetucci** | Cardiology | Faculty of Biology, Medicine and Health, The University of Manchester | The University of Manchester | No conflicts | England |
| **Patrick Mok** | Pharmacy (stroke and diabetes) | Doncaster and Bassetlaw Teaching Hospitals NHS Foundation Trust | Doncaster and Bassetlaw Teaching Hospitals NHS Foundation Trust | No Conflicts | England |
| **Rebecca Kuruvilla** | Clinical Pharmacology Internal Medicine | Department of Pharmacology and Therapeutics, Wolfson Centre for Personalised Medicine, The University of Liverpool, Liverpool | University of Liverpool | No conflicts | England |
| **Salim Elyas** | Stroke Medicine | The Academic Department of Healthcare for Older People, Royal Devon University Healthcare NHS Foundation Trust.  Department of Clinical and Biomedical Science, University of Exeter Medical School | Royal Devon University Healthcare NHS Foundation Trust.  University of Exeter Medical School | No conflicts | England |
| **Simon Ray** | Cardiology | Department of Cardiology, Manchester University NHS Foundation Trust, Manchester, UK. | The University of Manchester | No conflicts | England |
| **Sophie Harding** | Pharmacy | Velindre Cancer Centre, Velindre Road, Whitchurch, Cardiff | Cardiff and Vale University | No conflicts | Wales |
| **Sree Kondapally** | Cardiology | Cardiovascular Clinical Academic Group, Molecular and Clinical Sciences Research Institute, St. George's University of London, St. George's University Hospitals NHS Foundation Trust, London, United Kingdom. | St George’s University Hospitals | No conflicts | England |
| **Stefanie Lip** | Clinical Pharmacology  Internal Medicine  Pharmacogenomics | School of Cardiovascular and Metabolic Health, University of Glasgow, Glasgow, Scotland, UK. Queen Elizabeth University Hospital, Glasgow. | University of Glasgow | No conflicts | Scotland |
| **Tom AT Hughes** | Neurology | Neurology, University Hospital of Wales Healthcare NHS Trust, Cardiff, UK. | University Hospital of Wales | No conflicts | Wales |
| **Vicky Chaplin** | Pharmacy | Genomics Unit · NHS England | NHS England | No conflicts | England |
| **Vicky Stinton** | Genomics | North West GLH, The University of Manchester | The University of Manchester | No conflicts | England |
| **David Hargroves** | Stroke Medicine | Department of Stroke Medicine, East Kent Hospitals University Foundation Trust (EKHUFT).  Clinical lead for Stroke: EKHUFT & South East (Wessex, BOB, Kent, Surrey and Sussex) NHS England. Senior Clinical Adviser to NHS England GIRFT Stroke Programme. National Clinical Director for Stroke Medicine – NHS England. | East Kent Hospitals University Foundation Trusts | No conflicts | England |
| **Nicholas Greaves** | Vascular Surgery | Department of Vascular Surgery, Manchester Royal Infirmary, Manchester University NHS Foundation Trust, Manchester, UK | Manchester University NHS Foundation Trust | No conflicts | England |
| **Kerry Burke** | Vascular Surgery | Department of Vascular Surgery, Manchester Royal Infirmary, Manchester University NHS Foundation Trust, Manchester, UK;  Manchester Centre for Genomic Medicine, St Mary’s Hospital, Manchester University NHS Foundation Trust, Manchester, UK;  Division of Evolution, Infection and Genomics, School of Biological Sciences, The University of Manchester, UK | Manchester University NHS Foundation Trust;  The University of Manchester | Receives grant support from the NHSE Network of Excellence in Pharmacogenetics | England |

**Supplementary Table 2.** Comments received during the Consultation period by various organizations on the CERSI-PGx Guideline for *CYP2C19* Genotyping with Clopidogrel and Responses by the UK CERSI PGx writing committee

| **Organisation** | **Comments from organisation** | **Responses from Committee** | **Changes to the Guideline (if any)** | |
| --- | --- | --- | --- | --- |
| **The Medicines & Healthcare products Regulatory Agency (MHRA)** | The guideline developed by the UK CERSI-PGx consortium appears accurate, where mentioning current marketing authorisations by the MHRA (p4, p19) and is careful to note indications and where products are used off label (p12).  However, the presented evidence (and other guidelines) is at odds with the information in section 5.2 of the clopidogrel SmPC which presents the results of a small crossover study looking at active metabolite exposure and antiplatelet aggregation. The results were similar in the EM and IM group. Can the UK CERSI-PGx provide an explanation about this apparent discrepancy? | Thank you for the positive comment  There is a subtle but important difference between the objective of the Summary of Product Characteristics (SmPC) and the objective of a clinical guideline. The burden of the guideline is answering if there is sufficient evidence to suggest that we should actively recommend the use of clopidogrel in intermediate metabolisers (IMs).  There is no debate about safety (i.e. bleeding) at 75 mg/day. If we expect less bleeding in the IMs, this criterion of safety is met when using clopidogrel at 75 mg/day in this subgroup of patients. The UK CERSI-PGx has carried out our own analysis on clopidogrel pharmacokinetics and efficacy data, to understand the impact of the 150 mg/day dose in IMs. The 150 mg/day dose of clopidogrel is off-label, and will not influence the SmPC (at least for now). Additionally, we have not recommended the 150 mg/day dose.  Effectiveness is more challenging, with the literature somewhat divided on the effect IMs have on clinical outcomes. There is certainly a significant burden of literature demonstrating a poorer response, including some well conducted institutional reviews e.g. NICE analysis for stroke is compelling.  *Ex vivo* results are perhaps more consistent and show decreased platelet inhibition/ increased platelet activity for IMs and PMs using a variety of methods (the validity of each presenting its own challenge). Of note, C_max_ also appears to be a factor in platelet effects, and logically this would be more impacted by slower metabolism of clopidogrel to the active metabolite clopi-H4 than AUC is.  The SmPC of clopidogrel reports data on the pharmacokinetics and pharmacodynamics of clopidogrel from the following study: **Simon T et al. *Clin Pharmacol Ther*. 2011. doi: 10.1038/clpt.2011.127.**  The primary analysis showed no differences between CYP2C19 extensive metabolisers (EMs) and IMs in the exposure to the active metabolite clopi-H4 as well as in the extent of platelet inhibition (data reported in the SmPC as Mean Inhibition of platelet aggregation stimulated by 5 µM ADP). Interestingly, platelet inhibition results were not confirmed by using a different platelet reactivity assay, i.e., the vasodilator-stimulated phosphoprotein platelet reactivity index (VASP-PRI). This highlights the fact that platelet reactivity has not been fully validated as a measure of clopidogrel activity/efficacy and results may vary based on different tests adopted.  In addition, the paper included the results of a pooled analysis on a larger cohort of 396 subjects. This analysis included data from the 40 healthy volunteers (HVs) enrolled in the pharmacogenetic study reported above as well as data from HVs enrolled in 6 additional Phase 1 studies. The PK study population included 388 subjects for the loading dose (LD) and 353 subjects for the maintenance doses (MD), whereas the PD study population included 356 and 353 subjects in the LD and MD groups, respectively. The authors confirmed that the CYP2C19 metabolic activity is a major determinant of the exposure to the clopi-H4 active metabolite. Briefly, PK/PD data based on the *CYP2C19* genetic variability were in line with the results obtained in the pharmacogenetic study, indicating that *CYP2C19* poor metabolisers (PMs) had the lowest exposure to the clopi-H4 metabolite although a 30% reduction in the active metabolite formation was also observed in IMs. In particular, after 300 mg LD the ratio of the area under the curve (AUC) between IMs and EMs (IM/EM AUC ratio) was 0.77 (0.70, 0.85), p<0.001; and the PM/EM AUC ratio was 0.36 (0.30, 0.45), p<0.001. After 4 days with the 75 mg MD, the IM/EM AUC ratio was 0.72 (0.65, 0.79), p <0.001; and the PM/EM AUC ratio was 0.28 (0.22, 0.34), p <0.001 (Suppl. Table 7). The pooled analysis also showed reduced efficacy in the IMs and PMs *vs* EMs, although the magnitude of the negative impact on platelet aggregation was larger in PMs than IMs (Suppl. Table S8). However, doubling clopidogrel dose seemed to reduce PD variability in PMs (Table 4).  In contrast, the SmPC refers to a metanalysis of (only) 6 studies, including 335 subjects for the PK analysis and showed reduced differences between IMs and EMs than data reported by Simon T et al., 2011. The above data highlighting differences between IMs and EMs are not fully reported in the SmPC.  In conclusion, the PK section in the SmPC may need to be revised including the most updated evidence on this topic. | No  No | |
|  | Ticagrelor is not licensed in stroke/TIA in UK. This aspect could be more prominent in the guideline (mentioned in the points on page 12 but not in the table). The application to add this indication was initially submitted to the EMA but later withdrawn. At the time of withdrawal, the EMA was of the opinion that the benefits did not outweigh the risks**. It is important to note that the MHRA does not recommend off label use.** | More comprehensive information on ticagrelor use in stroke have been now included in Section 10, REGULATORY CONSIDERATIONS of the guideline. We have highlighted that the use of ticagrelor in certain indications is off-label (Section 10.1). | Yes, Section 10.1 | |
|  | **Minor points:**   1. Page 8 (paragraph 1- line 5): to avoid any confusion please refer intermediate and poor metabolisers in place of IMs or PMs. The team may consider using full wording or providing a glossary for abbreviations 2. Table 4 (page 8): under the UK CERSI-PGx recommendations there are recommendations on “likely” intermediate/poor metabolizers. The term “likely” is being used in the CPIC guidelines, but it hasn’t been defined in the CERSI-PGx guidelines for clopidogrel. It would be useful to define what that means as table 4 recommends action for these groups of patients. 3. The team should clarify under what circumstances the “Tier 2 Variants – Extended Panel” should be applied in the testing approach (p9-10) 4. It would be useful to clarify if the allelic frequency for ‘African’ provided in Table 3 (page 10) also apply to the Caribbean population, which is relevant to the UK. 5. If there are alleles which are not included in the table, and if appropriate, the CERSI team may consider adding a wording in this section that there may be other variants which have not been included in the CERSI guideline and in the panel test. It is noted that this is an area for which further research is necessary. We recommend that any novel data would be included in updated version of this guideline. 6. The CERSI guideline does not describe the limitations of the point of care testing (page 10). Whilst it is acknowledged that it is beyond the scope of this guideline to provide detailed information about all the different tests and the limitations of these tests, in this section CERSI team may consider as a minimum provide an appropriate reference. 7. Could the “Evidence Overview” section have some of the data tabulated for easier comprehension? 8. The link to the Plavix SmPC at Reference 4 appears to be broken and needs to be replaced. | 1. Abbreviations have been removed 2. The UK CERSI-PGx guidelines have mainly considered Tier 1 and 2 variants for the assignment of the phenotype and the clinical recommendations. These are all loss of function alleles (apart from **17*) and the phenotype is well defined. On the other hand, the “likely” intermediate/poor metabolizer phenotypes can occur if an extended panel of *CYP2C19* variants is used including variants that have been assigned reduced activity, like for example *CYP2C19*9*. In fact, the terminology appears in the 2022 update of the CPIC guidelines (**Lee CR et al.** ***Clin Pharmacol Ther***. **2022. doi: 10.1002/cpt.2526**). If these terms will be included in a pharmacogenetic report, the UK CERSI-PGx recommendations applied to IMs and PMs should be considered. The meaning of likely intermediate/poor metaboliser is explained in the note ‘l’ to current table 8. 3. At present most laboratories in the UK are providing pharmacogenetic testing for Tier 1 variants. However, considering the mixed genetic background of the UK population extended panels including other relevant variants to the UK population should be considered if available. 4. The *CYP2C19* allelic frequencies reported for ‘African’ in Table 3 refer to the biogeographical group Sub-Saharan African. These frequencies largely align with *CYP2C19* allelic frequencies in African-American and Afro-Caribbean biogeographical groups, as now reported in the updated Table 3. The source of data is the CYP2C19 frequency Table downloaded by the ClinPGx database (former PharmGKB) at <https://www.clinpgx.org/page/cyp2c19RefMaterials> 5. The UK CERSI-PGx included in Table 3 the allelic frequencies of the most well characterized *CYP2C19* allelic variants. As suggested, we included a sentence in the main text to refer the readers to the ‘CYP2C19 frequency Table’ available through the ClinPGx database. This table provides a more comprehensive view on the *CYP2C19* allelic variants, some of which are very rare or their function is still indeterminate (please refer also to the ‘*CYP2C19* Diplotype-Phenotype Table’ available at the same database). 6. The UK CERSI PGx is following the NICE guidance in this area, and we are consistent with the wording in that guidance. It is not the purpose of the guideline to specify which technology should be used to undertake genotyping. This needs to be left to the NHS and specific clinical situation 7. The Evidence Overview section was written taking into consideration the input of the clinicians, members of the writing committee. It is also important to note that the guideline will appear different when it is published in a journal. 8. The link to the Plavix SmPC was taken from the MHRA database, but it appears that the link is periodically changed and thus can be broken. At present also the link provided appears to be broken. We have included the link to the results from the search string, instead. This has been done for reference n. 4 (Clopidogrel SmPC) and reference n. 44 (Mavacamten SmPC). SmPCs can be visualized after accepting the MHRA disclaimer. | 1. Yes, Section 5.1.1 2. Yes, note ‘l’ to Table 8 3. No 4. Yes, Table 3 5. Yes, Section 6 6. No 7. No 8. Yes, references (n. 4 and n. 44) | |
| **National Institute of for Health and Care Excellence (NICE)** | Should the title be amended to: ' guideline for CYP2C19 genotype testing to guide clopidogrel use'? | The title was amended | Title page | |
|  | Recommendations re what to do on the basis of the CYP2C19 test results is very helpful. Guideline proposes extending testing outside of stroke & TIA, cost effectiveness analyses for testing in coronary artery disease and peripheral arterial disease are needed. Is it good value for money? | As reported in the Health Economic Evaluation section (Section 9.2), the available evidence in the clinical setting of Coronary Artery Disease support pharmacogenetic testing in the context of acute coronary syndrome, although the evaluations are based on health care systems outside the UK. The UK CERSI-PGx has highlighted the need of cost-effective analyses that are relevant to the NHS in the UK in the setting of coronary artery disease and peripheral arterial disease. This has been suggested as an area of further investigation (Section 12.1). | No | |
|  | Other than to outline when the testing is done, consideration of how this will be implemented is not outlined. Is the system ready and what other support may be provided? | The UK CERSI-PGx guideline provides state of the art evidence for the use of *CYP2C19* testing in clinical practice, the implementation process is beyond the scope of the guideline. | No | |
|  | The guidance is laid out in such a way that the actual recommendations are rather buried- is there a way to address this? The annexes are useful summaries- maybe state these upfront? | In the Executive Summary, the following sentence is included: ‘Summary guidance on a page is provided for each of the indications in Boxes 1-3’. | Yes, Executive summary | |
|  | The guideline is helpful, few comments:   - Please could the guideline team outline that the clopidogrel 150mg dose is off-label, I note they have done this for ticagrelor in stroke. - Prasugrel has a licensed 5 mg dose for adults <60kg and adult over 75 years, would it be helpful to also reference this dose as an option in the table or bullets below as there may be an interpretation that the guideline is only recommending the 10mg dose? - I think potentially the layout could be clearer as I agree it is hard to find the recommendations, I think having the annexes upfront would be helpful as I think they would be easy to miss under all the references - I do understand why this point is made throughout the guideline *‘In the absence of available relevant pharmacogenetic information or testing, the current best practice clinical guidelines should be followed’* however, for example for CAD the guideline advises that in poor metabolisers loading doses should be avoided but then advises that if this information isn’t available  follow the current best practice which in some situations is to give a loading dose. I’m not sure there is a solution but just wanted to highlight how this may concern prescribers/ patients who don’t have that information. | - The use of clopidogrel 150 mg once daily has been clearly stated as off-label - This has been amended. - Please see comment above. - This comment clearly highlights the relevance of pre-emptive *CYP2C19* genotyping to choose the best dose of clopidogrel or the best treatment option for the patients. Therefore, genotyping should be widely implemented regardless the clinical indications any time clopidogrel is the drug of choice. | Yes, Section 7.4  Yes, Section 7.2  Yes, Executive Summary.  No | |
|  | Page 20: HEALTH ECONOMIC EVALUATION - Stroke and transient ischaemic attack’: Genedrive is stated as the preferred POC test from the NICE guidance based on cost. However, the committee did note that the estimated cost per test for Genedrive is less than for Genomadix Cube and concluded that Genedrive was its preferred point-of-care test but this wasn’t’ the only reason for this preference. Please see text from the full guidance below (section 3.15):  “The committee also noted that several features of the Genedrive test could offer advantages over the Genomadix Cube (see [sections 2.12 to 2.15](https://www.nice.org.uk/guidance/dg59/chapter/the-diagnostic-tests#genedrive-cyp2c19-id-kit)). For example, its reagents do not need to be stored in a freezer and it can detect several additional alleles including those that occur in greater frequency in some ethnic groups (see [section 3.8](https://www.nice.org.uk/guidance/dg59/chapter/committee-discussion#less-common-loss-of-function-alleles)). The committee also noted that the estimated cost per test for Genedrive is less than for Genomadix. So the committee concluded that Genedrive was its preferred point-of-care test.”    When talking about laboratory testing in this section, Agena Bioscience iPlex MassARRAY platform is referenced. While this was used in the economics work for the NICE guidance to provide some model parameters, the consideration and recommendation was for lab based testing more broadly, so we were a bit concerned this may give some indication that NICE were favouring this particular platform. Could this reference to the Agena system perhaps be removed from this section. | This was amended as suggested  Now removed and referred to as lab testing. | Yes, Section 9.1  Yes, Section 9.1 | |
| **British Cardiovascular Society (BCS)** | We note that the ‘CYP2C19 for Clopidogrel Guideline Group’ authorship has wide representation which is welcomed, with an appropriate mix of cardiologists (many of whom are existing BCS members), stroke physicians, pharmacologists, pharmacists, health economics and genomics experts. We applaud the authors in their attempts to extend the guidance for CYP2C19 genotype testing to guide clopidogrel use beyond that of ischaemic stroke or transient ischaemic attack (current offering from NICE DG59). The guideline is comprehensive, well-written and has a detailed evidence base.  In regard to the indications for testing section, is it justified to extend this guidance to peripheral arterial disease when no economic evaluations are available identified for CYP2C19 testing in relation to the use of clopidogrel in peripheral arterial disease? By contrast, it seems very reasonable to include it for coronary disease, acknowledging that more cost efficacy work is needed, in particular data relevant to UK practice.  We agree with the cited NICE view regarding the “likely shift from reactive to pre-emptive testing”. We would encourage the authors to consider commenting more if possible on the logistics of testing in the NHS. The suggestion to perform laboratory rather than POC CYP2C19 testing is in keeping with NICE but discrepant with other consensus statements (JACC CVI 2024; 17:2639-63) which have suggested that POC PLT testing should be encouraged if clopidogrel monotherapy is being contemplated among patient groups known to be associated with higher levels of clopidogrel resistance. We would also like to see some more detail where possible (or include as a limitation if data are sparse) on the health economics of POC testing for everyone on clopidogrel DAPT.  Generic prasugrel is already available for some and new anti-platelet drugs are in development.  The interesting clinical question is why given 30% hypo-respond to clopidogrel relatively few clopidogrel hypo-responders exhibit STEMI or other MACCE. In a South Korean population CYP2C19 LOF seemed not to influence the incidence of MACCE (from SMART CHOICE trial, acknowledging that small numbers were genotyped).    In the executive summary, we learn that an offering will be made on ‘appropriate laboratory turnaround times’ but we could not find these and we do think they would be a useful addition.    In terms of logistics, it may also be worth commenting that there are already established UK centres already in place offering NHS funded (laboratory rather than POC) testing for CYP2C19 genotype to guide mavacamten prescribing. There are notable transatlantic differences in the guidance for CYP2C19 testing among patients being considered for treatment with mavacamten, a novel myosin inhibitor; unlike in Europe and the UK, CYP2C19 testing is not routinely performed in the US for this indication. Table 4 offers a very helpful overview of the existing guidance and is helpful to non-experts in providing a more global overview of existing practice and guidelines.    We welcome the recommendation on better communication with primary care, as well as the importance of ensuring the result is easily visible. CYP2C19 variant status has implications for the prescription of many other drugs including PPIs and commonly prescribed antidepressants. We note that this has been incorporated into a section on ‘OTHER CONSIDERATIONS’ but believe it deserves highlighting  with a separate heading in its own right. We would also recommend a small addition within this section on the importance of educating patients on their metaboliser status.  The future research ideas section is very well received.    Overall, the BCS Guidelines and Practice Committee is supportive of this document. | Thank you for the positive comments and support.  Clopidogrel is first line therapy for secondary prevention of peripheral arterial disease (PAD), and thus widely used in this indication. As highlighted in the guideline patients with PAD are likely to have atherosclerotic arterial disease in both the coronary and cerebral circulations, and thus clopidogrel is potentially protective against cardiac and cerebrovascular ischemic events. The mechanism of action of clopidogrel in this condition is the same as in coronary artery disease (CAD), as are the pharmacokinetics. We have highlighted the need for health economic analyses.  The logistics of the implementation is beyond the remit of this guideline. The UK CERSI-PGx focused on the clinical utility of genotyping. Even though POCs for *CYP2C19* genotyping have been validated, NICE recommendation in the stroke indication is to use a laboratory-based test when available. The implementation of the guidance is now under consideration of the NHS, with pilot projects started in the country and a national genotyping scheme launched in Scotland  (<https://markets.ft.com/data/announce/detail?dockey=1323-16944139-7LT0JRVAG5SCJMKVN0T5QC4O52>).  Regarding platelet function tests (PFTs), we are aware that in the 2024 update of the International Consensus Statement on Platelet Function and Genetic Testing in Percutaneous Coronary Intervention (**Angiolillo DJ et al., *JACC Cardiovasc Interv*. 2024 doi: 10.1016/j.jcin.2024.08.027**) the use of POC tests over laboratory-based tests is suggested. However, PFTs have not been fully validated to assess clopidogrel efficacy. The Consensus Statement indeed provides different cut-off points for different tests to define ‘high’ and ‘low’ platelet reactivity. These values can serve as a useful reference for implementation of PFTs in practice. Notably, the Consensus Statement also highlights the limitations of these assays, including the uncertainty on the optimal timing of testing in relation to the PCI, inter-assay and intra-patient variability of readouts, difficulties in using these tests in the context of ACS and during de-escalation from prasugrel/ticagrelor to clopidogrel. We have included a statement in the research recommendation about the need for further evaluation of PFTs in association with genotyping tests.  This is an interesting observation, and it is complex to address this point. However, we should keep in mind that the overall better outcome observed in patients after PCI may be well associated with the advances in stent design (drug eluting stent) as well as in intravascular imaging techniques used during stent implantation. This has led to a paradigm shift in the field, that is to protect patients from the risk of bleeding more than thrombosis. This is also related to the use of ticagrelor and prasugrel as first line antiplatelet agents. However, in this context, clopidogrel remains a widely prescribed drug, especially in patients at high risk of bleeding and in the elderly. The SMART CHOICE 3 trial confirmed the long-term efficacy of clopidogrel *vs.* aspirin in secondary prevention, in patients that have completed the DAPT cycle. On the other hand, it is difficult to evaluate the relevance of pharmacogenetics in this trial. Only 731 patients out of 2752 in the clopidogrel arm were genotyped (Suppl. material, Fig S6). Moreover, the comparison has made between rapid/normal metabolisers vs intermediate/poor metabolisers (**Choi KH, et al. 2025, Lancet. 2025. doi: 10.1016/S0140-6736(25)00449-0**). It is possible that the effect of clopidogrel is diminished in poor metabolisers *vs*. rapid/normal metabolisers and that these subjects would benefit from aspirin treatment more than clopidogrel. A recent meta-analysis, including also the results from the SMART CHOICE 3 trial, has confirmed the higher benefits of clopidogrel *vs* aspirin in the longer term (**Valgimigli M et al., 2025; Lancet. 2025. doi: 10.1016/S0140-6736(25)01562-4**). The authors highlighted that the genetic variability can be more relevant in the acute setting when using clopidogrel, in comparison to the long-term use. However, this is difficult to evaluate considering that exploratory *CYP2C19* genotyping was only included in the SMART-CHOICE 3 trial.  Please refer to page 11 of the guidelines, in which we stated that acceptable laboratory turnaround time is 5 days or less. We deliberately did not recommend any specific assay, considering the rapid advances in the field of genotyping thus leaving the choice the laboratory.  We have included a sentence that *CYP2C19* genotyping tests is nationally available for the prescription of mavacamten. As mentioned above is beyond the remits of this guideline provide details on the implementation of genotyping.  Specific guidelines on the use of *CYP2C19* genotyping for the optimisation of other drugs will be developed, hence the limited information provided in this guideline. In the future, we may include links to the other guidelines related to *CYP2C19*. About patients’ education we shared the guideline with two patients’ representatives, part of the PPIE group established for the delivery of work package 5 of the UK CERSI-PGx PROJECT. A lay summary has been included in the supplementary file to help patients to understand the relevance of genetic variability on the benefits/risk profile of clopidogrel. | No  No  No  Yes, Section 12.3  No  No  Yes, Section 11  No | |
| **British Cardiovascular Intervention Society (BCIS)** | Clinical experience is that there is a disconnect between in vitro and in vivo effects. We certainly do not follow what you are suggesting in the guideline. | The UK CERSI-PGx is aware that pharmacogenetic testing before clopidogrel prescription is not routinely performed in the UK. However, there is robust evidence to support the implementation of testing in the cardiovascular setting (refer to page 6, Coronary artery disease sub-section). It has been consistently shown that the efficacy of clopidogrel is reduced in patients who are intermediate or poor metabolisers. Please note the high frequency of these phenotypes in biogeographical groups as East and Central/South Asians (> 50%) and Afro-Caribbeans (~ > 35%) (newly added Table 4). | No | |
|  | Many patients receive clopidogrel for coronary and stroke indications. Genotyping for clopidogrel responsiveness has not been useful in predicting adverse cardiac events. Please refer to the following trials: TRIGGER-PCI 2012, TALOS-AMI 2021, TALOS-AMI 2024. | We considered the literature suggested. The papers cited assessed de-escalation from prasugrel or ticagrelor to clopidogrel in stable patients at least one month after PCI. They found no significant difference in the primary endpoint; however, some studies reported a lower rate of bleeding in patients treated with clopidogrel. This finding may not be surprising in this population, as with newer-generation stents, endothelization can occur within 30–40 days. In addition, the risk of bleeding after PCI is highest during the first 30 days. The optimal timing for performing genetic testing to guide clopidogrel therapy is at the time of PCI, in order to immediately identify the most appropriate antiplatelet regimen. This was clearly demonstrated by **Claassens and colleagues (doi: 10.1056/NEJMoa1907096)**, who showed that in patients undergoing primary PCI, a *CYP2C19* genotype-guided strategy for selecting oral P2Y12 inhibitor therapy was non-inferior to standard treatment with ticagrelor or prasugrel at 12 months with respect to thrombotic events and resulted in a lower incidence of bleeding. In addition, we have quoted the results associated with TAILOR-PCI in the evidence overview, and in particular the fact that the pre-specified endpoint just missed significance, but there was a significant difference when all events were evaluated over the follow-up period, and subsequent meta-analysis have also shown the value of genotype-guided use of clopidogrel.  As mentioned above, the UK CERSI-PGx is aware that pharmacogenetic testing before clopidogrel prescription is not routinely performed in the UK (please refer to Section 3.2, Coronary Artery Disease). The purpose of this guideline is to encourage the use of pharmacogenetic testing when clopidogrel is the drug of choice in any specific clinical indication. | No | |
|  | We don't agree with the genetic testing approach. For heterozygotes for the loss of function allele, there is so much overlap with normals in terms of clopidogrel response that a phenotypic test would be needed to accurately find out who is responding and who is not to the therapy. Furthermore, the only commonly used frontline assay for platelet response is VerifyNow, which is not great at testing response to clopidogrel. TEG6s would be a much better option. Please consider a recent review attached with full arguments and a couple of relevant papers. There is a section in the review about why genotyping isn't a good option. In summary, genotyping is not a good enough predictor of phenotypic response alone    References attached:  Khanna V, Hobson A, Mikael R, et al. Does the VerifyNow P2Y12 assay overestimate "therapeutic response" to clopidogrel? Insights using short thrombelastography. *Thromb Haemost*. 2014; 111: 1150-1159. doi: 10.1160/TH13-10-0856.  Khanna V, Armstrong PC, Warner TD, Curzen N. Prostaglandin E1 potentiates the effects of P2Y12 blockade on ADP-mediated platelet aggregation in vitro: Insights using short thromboelastography. *Platelets*. 2015; 26: 689-692. doi: 10.3109/09537104.2014.1001832.  Elserwey A, Jabbour RJ, Curzen N. Does one size really fit all? The case for personalized antiplatelet therapy in interventional cardiology. *Future Cardiol*. 2024;20: 499-515. doi: 10.1080/14796678.2024.2384217. | The UK CERSI-PGx does not entirely agree with the suggestion that genotyping is of no value based on the studies mentioned especially when platelet function testing is fundamentally flawed by the use of several different assays, TEG, Verify Now, Flow Cytometry (CD62p or CD40 ligand) whole blood impedance, standard aggregometry and many more. Given that there are so many mechanistically different assays, one might suggest that none of them is effective explaining the disconnect. None of these assays take into account the complex interactions that occur in response to a host of platelet agonists including Collagen, Epinephrine, vWF, ADP or arachidonic acid.  Flow cytometry may well be better but it is expensive, and not practical. The methods to assess *ex vivo* platelet responses are suboptimal, explaining in part the lack of correlation between the genotype and the phenotype. We would therefore agree with the comment about VerifyNow, but other tests also have their limitations. What is true which is seen using a variety of assays is that there is a bimodal distribution of platelet responses to Clopidogrel not seen with Ticagrelor and Prasugrel. If high platelet reactivity (HPR) is considered important, although measured using a variety of platelet function tests, we can underline that HPR prevalence is significantly lower in Ticagrelor treated patients versus Clopidogrel treated patients. It follows then that the significantly lower rate of ischaemic events in Ticagrelor treated patients seen in PLATO is due to its consistent homogenous inhibitory effect compared to the much more heterogenous response seen in Clopidogrel treated patients. There is conflicting evidence in this area at present that the previous negative studies are compromised by the simplicity of using one assay which in itself is not ideal.  In addition, patients most likely to have resistance to clopidogrel are the poor metabolizers, because they will have no active metabolite, and they are readily identified by genetic testing. The intermediate metabolizers are a heterogeneous group and more research is needed to understand the best way to phenotype them and prescribe the correct antiplatelet treatment regime for them.    In conclusion, the main advantage of genotyping is that it can be performed pre-emptively and the right drug/dose can be chosen without exposing patients to inappropriate therapies. It is also important to remember that the quality assurance processes for genotyping are much better developed than for platelet function testing which allows scalability without inter-lab variation. However, other factors may contribute to the pharmacological response to clopidogrel in patients with LOF alleles as well as in normal metabolisers. Among these, the validation of a platelet function test that can measure clopidogrel response could be a useful and complimentary tool to provide better care, pending the right validation. This research gap has been highlighted in the research recommendation section. | Yes, Section 12.3 | |
| **Vascular Society** | No response received |  |  | |
| **British Pharmacological Society (BPS)** | Thanks for the opportunity to feedback on the CERSI-PGx guideline. Responses from the clinical committee and expert comments below.  You asked specifically for comments on the ‘indications for testing’ section. My view is that you could add strength to your recommendations should you choose to. Currently (bottom of page 7) you say that people ‘*should be considered’* for pharmacogenetic testing. In the executive summary you state that ‘*in these patients [poor/intermediate metabolisers], the clinical effectiveness of clopidogrel is reduced or absent’.* If the view of CERSI-PGx is that this is clinically meaningful (which seems to be the sentiment from the executive summary) then should CERSI-PGx not be recommending testing for *all* patients where an alternative effective therapy exists? Indeed, in the section below (page 8) where indications are gone through line by line, it seems that in all indications you recommend testing. The strength of the recommendation is therefore (to my eyes) not consistent between opening para of the recommended indications section and the more detailed text that follows.  Final minor comment is that there could be a bit of consistency in language on testing. In some sections you say ‘testing to identify clinically relevant CYP2C19 variants’, sometimes it is ‘testing for CYP2C19 variants’ and in others ‘CYP2C19 genetic testing’ and others ‘testing for CYP2C19’. There is presumably some difference in testing for ‘clinically relevant’ vs ‘all variants’. I would suggest one phrase is chosen and applied to the document.  My main concern from a primary care perspective is that it would help if it could provide explicit guidance on what primary care should do. If hospital are requesting a genetic test prior to commencing clopidogrel, it seems to me that the options would either be that prescribing/PGx testing is undertaken by the hospital and not delegated to the GP, or clear guidance is offered to the GP on what steps need to be taken (although I suspect there will be kickback from GPs in that circumstance, due to issues around funding etc). I suspect that the number of times a GP initiates clopidogrel in primary care without secondary care recommending it may be low, but some text to support this process may be helpful - indeed, if access to genetic tests is not readily available through primary care, should the recommendation be NOT to prescribe this drug in primary care as a result?  I am also unclear what "RCGP position statement" is being referred to in the guidance - I know RCGP have one on consumer genetic testing, which doesn't seem entirely relevant in this context (although of course raises issues about having suitable expertise to interpret the test), and I think RCGP also raised concerns about increased prescribing of clopidogrel more generally (but I don't think that was specifically relevant to PGx issues); I am unaware if there is a specific position statement in relation to medically indicated PGx testing or clopidogrel testing in particular?  There is some inconsistency though in how it expresses recommendations between indications in the main text of the guideline, which I think is a little confusing i.e. "no action required" vs "prescribe clopidogrel" vs "no pharmacogenetic informed action required. Follow the recommended prescribing guidelines...". This is then expressed more consistently for all indications in the appendix as "No pharmacogenetic informed action required." [See images below]  Will they be seeking endorsement from appropriate specialty groups e.g. British Association of Stroke Physicians? At St George's they are not opting for dipyridamole (I did ask them why in the guideline development and they didn't respond - there are reasonable arguments both ways I think, but they didn't provide their justification to me). | The wording has been changed to make it more consistent between the different sections.  Harmonisation throughout the text has been performed, including mainly the wording pharmacogenetic testing.  *CYP2C19* genotyping will largely be initiated in secondary care. A specific section on communication between secondary care and primary care is included. This was prepared with the input of two general practitioners, members of the writing committee. In addition, the CERSI-PGx guidelines highlighted that testing is required for patients. It is not for patients who are on clopidogrel unless they have some clinical reasons. So, we do not expect GPs to request pharmacogenetic testing routinely, although in some circumstance they may refer patients on clopidogrel for genotyping to secondary care physicians. It is also possible that in exceptional circumstances, GPs may want to undertake *CYP2C19* testing, and this may become more commonplace in the future.  The RCGP position statement became available after the guideline was accepted for publication and can be accessed at the following link <https://www.rcgp.org.uk/representing-you/policy-areas/genomic-position-statement>.  Harmonisation throughout the text has been performed, including only the wording ‘no pharmacogenetic informed action required’ referring to the rapid and ultra-rapid metabolisers and the normal metabolisers. Same wording is now included in the current Table 8 that provides a comparison of recommendations among different international pharmacogenetic consortia.    The guidelines has been shared with the British and Irish Association for stroke Physician. It is important to note that we are not seeking endorsement from any organisation, but rather their views. Dipyridamole is the MHRA licensed option if clopidogrel cannot be prescribed, but we understand that its use may be lower because of issues with tolerability. Ticagrelor is an alternative choice but we have highlighted that its use in stroke is off label. | Yes  Yes  No  Yes, Section 5.4  Yes , throughout the document  No | |
| **Royal College of General Practitioners (RCGP)** | No response received |  |  | |
| **UK Clinical Pharmacy Association (UKCPA)** | **Summary of comments**  The CERSI-PGx guideline for clopidogrel is a comprehensive, clear and well-structured, evidence-based resource that can be used by non-PGx experts in clinical practice. It provides a thoughtful analysis of contemporary evidence linked to UK practice, addressing pertinent topics such health economics, phenoconversion, research recommendations, impact of using alternative agents on efficacy and safety, and the timing of the genotyping in the timeline of the patient. | Thank you for the positive comments and support. | No | |
|  | **Alignment with UK practice and guidelines**:  We note the group membership represents some of the following groups, but further consultation with NICE, NCGS, and other prescribers including GPs as likely long-term prescribers would be welcomed to support uptake and alignment of the affected areas. We note that all possible options are discussed, but in context of the recommendations for non-normal metabolisers ideally there would be a reciprocal recommendation agreement between the organisations to avoid mismatches in different PGx profiles and scenarios, and the NICE and NCGS guidelines will be updated accordingly.  **Recommendation for clopidogrel 150 mg (+ 600 mg loading dose)**  We have concerns that this practice is new for the UK and not aligned with established practice, including recommendations in guidelines from NICE or the National Clinical Guideline for Stroke (NCGS). It was helpful to see the discussion of the evidence behind this dose. It would be good to emphasise more this is off-label and refer to local formularies when it is mentioned, although we note it is discussed briefly in some parts of the guidance. The paragraph comparing bleeding risk from the higher dose could be paraphrased as it is currently unclear. A more in-depth discussion on PK/PD of the off-label dose would be welcomed to support prescribers in decision making, including a discussion on the utility of platelet reactivity testing for example as the evidence is mixed (Krishna 2012) with patients remaining resistant in doses up to 2400 mg. The CPIC guidance (Mega 2011, and Horenstein 2014 primary sources) to recommend 225 mg for those IM patients who have no other options and 300 mg in diabetic IM but ultimately omits these doses from the recommendations.  **Recommendations for aspirin and dipyridamole**  This is supported by evidence which we support as an alternative but noting it is found at odds with NCGS and UK practice in some sites. We have concerns that this practice is also not aligned with established practice, or the current NCGS.  In more detail, when comparing the guidelines, in the NCGS in section  *“5.6 Antiplatelet treatment” it states the following: “For long-term prevention of vascular events in people with ischaemic stroke or TIA without paroxysmal or permanent atrial fibrillation:*  ** clopidogrel 75 mg daily should be the standard antithrombotic treatment; * aspirin 75 mg daily should be used for those who are unable to tolerate clopidogrel”*  In the “Evidence to recommendations” it states the following:  *“Comparative trials such as CAPRIE (CAPRIE Steering Committee, 1996), ESPRIT (ESPIRIT Study Group, 2006) and PRoFESS (Sacco et al, 2008) show that aspirin plus modified-release dipyridamole and clopidogrel monotherapy are equally effective, with both options superior to aspirin monotherapy”.*  Moreover, two pilot sites involved in the pilot clopidogrel study opted to use aspirin monotherapy for long-term antiplatelet treatment in PM and IM. | The UK CERSI-PGx guidelines have been developed with the contribution of clinicians who are the main prescribers of clopidogrel. This includes neurologist and stroke physicians, cardiologists and vascular surgeons. In addition, the writing committee included two general practitioners to define a clear communication pathway between secondary care and primary care. The guidelines have been shared with relevant National Societies, including NICE and the British and Irish Association of Stroke Physicians.  In the revised version of the guideline, the UK CERSI-PGx has highlighted the off-label use of 150 mg/day of clopidogrel and removed the recommendations from Tables 5-7 and Boxes 1-3, referring the users to the revised section 7.4 ‘Use of alternatively dosed clopidogrel in CYP2C19 intermediate metabolisers’.  The UK CERSI-PGx guidelines recommend the use of aspirin 75 mg once daily and dipyridamole MR 200 mg twice daily as first option in patients that are unable to metabolise clopidogrel properly. This according to the licensed indications of dipyridamole and supported by randomized clinical trials. However, from clinical practice, we are aware that up to 30% of patients do not tolerate dipyridamole, with headache being a major cause of early discontinuation. Therefore, these patients will continue with aspirin monotherapy. | No  Yes, Section 7.4  No | |
|  | **Indication, Testing and interpretation:**  The guidance and summary tables are easier to read than some international PGx guidelines. We had a concern that recommendation to genotype CYP2C19 for all clopidogrel patients can potentially be misinterpreted as implying universal testing availability, but acknowledging that commissioning availability of the test and its regulation is discussed in a few areas of the document. | Recommendations for CYP2C19 genotyping is mainly for new patients, for whom clopidogrel is the considered treatment option, although in some circumstances it may be necessary to request the pharmacogenetic test for patients already in treatment. | No | |
|  | **Ethnicity and allele frequency:**  Considering the large absolute numbers of patients requiring to be genotyped at population level, it may be useful to summarise the allele frequencies relevant to UK populations which may need to be more detailed and precise to support decision making that is equitable and inclusive of those with rarer alleles, that may now present more often due to the numbers tested, and discussing in context with the sequencing platforms that prescribers have available. For example, if we expect to genotype 150,000 patients who are indicated for clopidogrel in stroke in the NHS annually, we should we consider presenting rarer AF at lower decimal points to highlight potential inequalities e.g. Up to 5 or 6 decimals to support the UK population-wide genotyping vision. However, I appreciate this can make the table more crowded, so this could be an appendix or a summary in the main CERSI-PGx portal. Converting to percentages (%) will condense the table and make it more user-friendly to those not familiar with AF or have a table legend explaining the figures. The recent UKBB WGS data publication may support with this. | Table 3 has been updated including allele frequencies relevant to the Afro-Caribbean population which is relevant to the UK. In addition, a newly added table 4 including the frequencies of different phenotypes has been added, to provide a quick reference to clinicians of the relevance of the *CYP2C19* pharmacogenetic testing in different ethnicities. | Yes, Tables 3-4 | |
|  | **Other editorial and formatting feedback:**  A contents table after the first page would be useful considering the length of the paper.  Page 8 guidance on integrating testing in pathways, need to define IM and PM (refer to table 1)  Anti-aggregate and antiplatelet terms used - stick to antiplatelet  Tables in pages 11-14 could potentially be different colours to enable users and emphasise the different indication  In table 4, the poor metaboliser row is perhaps one of the most crucial ones – suggest swapping it with the ‘likely poor’ row to ensure it is on the main page of the table, unless the table can be shrinked slightly.  Moreover, we appreciate the use of footnotes due to lack of space with duplicate information e.g. a use prasugrel or ticagrelor at standard dose if no contraindication – but noting a risk that first-time users may find it confusing or complex to follow. Consider splitting into themes. | The guideline will be submitted to a journal and will be formatted according to the journal style.  Reference to Table 1 has been now included.  The term antiaggregant was used in the comparative table, including recommendations from other international pharmacogenetics consortia (current Table 8). As reported in the Disclaimer, exact wording from these guidelines is included in the table, like the term aggregation and antiaggregant. In the entire document, the term antiplatelet has been used.  Tables will be formatted according to the Journal style, upon publication.  As suggested, the poor metaboliser row has been swapped with the ‘likely poor’ to be on the main page.  The purpose of this table is mainly to rapidly compare the UK CERSI-PGx recommendations (presented in a synthetic form) to the recommendations of other consortia. The users should refer to the main tables or annexes when prescribing clopidogrel. Footnotes seem to be appropriate. | No  Yes, Section 5.1.1  No  No  Yes, Table 8  No | |
| **The Royal Pharmaceutical Society (RPS)** | The Royal Pharmaceutical Society (RPS) welcomes and supports this work, recognising it as a positive step forward in the integration of pharmacogenomics into clinical care. It highlights an area where pharmacists are well positioned to take a leading role. We believe this direction not only strengthens the role of pharmacy within multidisciplinary teams but also ensures patients benefit from the full breadth of pharmacist’s clinical expertise.  Whilst we are not commenting on this specific test, due partly to the fact that each of the devolved nations may commission pharmacogenomic services differently, we will continue to highlight how pharmacists can take a leading role in shaping and delivering these services | Thank you for the positive comments and support. | No | |
| **British and Irish Association of Stroke Physicians (BIASP) -** Response approved by the BIASP Clinical Standards, Scientific and Executive Committees | BIASP support the goal of personalised antithrombotic therapy to reduce recurrent major adverse cardiovascular events in patients with recent ischaemic stroke or TIA. However, while CYP2C19 genotype testing is a promising tool, we believe that its routine use in stroke care is premature without stronger evidence from randomised controlled ‘test and treat’ trials. Indeed, this concern is acknowledged in the document *(“…the absence of studies to provide direct evidence on the efficacy of genotype testing*”, page 20).  We summarise some key concerns as follows: | Our guideline is consistent with the NICE guidance on the use of pharmacogenetic testing for clopidogrel in the stroke and TIA indications. | No |  |
|  | 1. **The randomised clinical trial evidence is extremely limited**   The CHANCE-2 trial, often cited in support of genotype-guided therapy, was conducted exclusively in Chinese patients with minor stroke or TIA. It showed only a modest absolute risk reduction (~1.6%) in recurrent stroke with ticagrelor–aspirin vs clopidogrel–aspirin in CYP2C19 loss-of-function (LOF) carriers (**Wang Y*et al*. N Engl J Med 2021; doi: 10.1056/NEJMoa2111749**). However, we are not aware of any large RCTs demonstrating that genotype-guided therapy improves outcomes in diverse, non-Asian populations or in patients with moderate-to-severe stroke. Until further randomized clinical trials are conducted, it is not possible to confirm reductions in the incidence of major adverse cardiovascular events by undertaking genetic testing. | The UK CERSI-PGx has outlined the evidence available for the use of clopidogrel in stroke/TIA, and this is consistent with the NICE diagnostics guidance (**DG59 – published 31 July 2024**) and with the Health Technology assessment commissioned by NICE (**Carroll J et al.** ***Health Technol Assess*. 2024. doi: 10.3310/PWCB4016**). NICE has recommended the use of *CYP2C19* genotype testing to determine if clopidogrel is a suitable antiplatelet agent for people with a recent ischaemic stroke or a transient ischaemic attack. Running RCT in this area is likely to be logistically very difficult due to a large number of participants needed given the small prevalence of LOF allele in Caucasian populations. An issue of equipoise may also arise, and thus ethics committees may question the use of a potentially ineffective treatment (i.e. Clopidogrel) in LOF allele stroke/TIA patients and compare it with other known effective treatments. | No |  |
|  | **2. Ethnic and population variability limit the generalisability across populations**  The prevalence of CYP2C19 LOF alleles varies significantly by ancestry, being highest in East Asians (~60%) and lowest in Europeans (~25%). This raises questions about the cost-effectiveness and clinical relevance of routine testing in populations with lower allele frequencies, such as in the United Kingdom and Ireland. It will be difficult to justify the expense of repeating the CHANCE-2 trial for populations that do not have a high incidence of LOF alleles (e.g., Caucasians), as many more subjects must be screened to find those carrying CYP2C19 LOF alleles (**Wu AHB et al. *J Appl Lab Med* 2025;** **doi: 10.1093/jalm/jfaf041)** | This point creates a counter argument to point 1. If the argument is that we cannot run an RCT in a mainly Caucasian population given the low prevalence of LOF allele then surely we have to find a pragmatic solution for a recognised biological problem. Furthermore, what about the towns and cities with a higher proportion of non-Caucasian populations who are by definition at higher risk! | No |  |
|  | **3. Biological plausibility does not provide evidence of clinical benefit**  While the pharmacogenetic rationale is sound, because clopidogrel is a prodrug requiring CYP2C19 activation, this biological plausibility does not guarantee improved outcomes with genotype-adjusted therapy. While observational studies and *post hoc* analyses suggest increased ischaemic stroke recurrence risk in LOF carriers, these are not substitutes for prospective, adequately powered RCTs. It is possible the LOF genetic variants have other effects beyond clopidogrel metabolism that could influence recurrence. It is difficult to determine whether the risk of stroke is because of CYP2C19 carrier status on clopidogrel metabolism or if it is due to other biological pathways that are independent of clopidogrel. Supporting this idea, the ACTIVE-A (Atrial Fibrillation Clopidogrel Trial With Irbesartan for Prevention of Vascular Events) found no interaction between the effect of clopidogrel and LOF carrier status on vascular outcomes (**Paré G et al., N Engl J Med. 2010 doi: 10.1056/NEJMoa1008410**) Similar findings were reported in a meta-analysis of 4 placebo-controlled RCTs (N=11,477) (**Holmes MV et al., JAMA. 2011. doi: 10.1001/jama.2011.1880**) Although the CYP2C19 enzyme has the most effect on the pharmacokinetics and pharmacodynamics of clopidogrel, there are other enzymes that are involved with this metabolism including CYP1A2, CYP2B6, CYP2C19, and CYP3A4/5 (**Wang Y et al. N Engl J Med. 2013 doi: 10.1056/NEJMoa1215340**). Other factors including age, diabetes, body mass index, drug interactions (e.g., with PPIs), and adherence can also influence the platelet response to clopidogrel. Recent data indicate that CYP2C19 genotype is only one factor contributing to antiplatelet drug response: not all CY-P2C19 LOF carriers or noncarriers have reduced platelet inhibition. Thus CYP2C19 genotype might not be a reliable predictor of the platelet aggregation response to clopidogrel (**Pereira NL et al., Circulation. 2024. doi: 10.1161/CIR.0000000000001257**). | Again the second point in BIASP’s response makes this argument not relevant and means we will have to adopt a pragmatic approach regarding *CYP2C19* testing rather than trying to source funding for a large RCT that could prove very difficult to deliver  We have also included the need for further research in the use of platelet function testing, and whether this should alone or in combination with genotyping. | No |  |
|  | **4. Implementation and resource use considerations**  Genotype testing introduces logistical complexity, including turnaround time, cost, and the need for specialist testing equipment and clinician education. In a health system with pharmacogenomic testing must be weighed against other priorities. Of note, CHANCE-2 did not report a health economic analysis | As mentioned above (point 1), NICE outsourced an economic analysis that showed cost effectiveness for both lab and point of care testing in stroke patients. | No |  |
|  | **5. Alternative strategies that do not require genetic testing exist**  For many patients, aspirin monotherapy, aspirin plus dipyridamole, or ticagrelor might be reasonable alternatives without the need for genotyping. The incremental benefit of tailoring therapy based on CYP2C19 genotype — especially when any absolute risk reduction is likely to be small — remains uncertain | The UK CERSI-PGx agrees that ticagrelor could be potentially used in the acute phase for all patients with TIA and minor stroke, but its use is not recommended by the available guidelines in non-minor stroke. We have highlighted the need for more research in this area. Although ticagrelor will become generic in 2026 and thus could potentially be used in minor stokes and TIA, there is a lack of long-term safety data for ticagrelor in stroke patients; this is highlighted in our guideline. We are also aware that many patients do not tolerate dipyridamole well and discontinue it. | No |  |
|  | **Specific comments on the document:**  **Page 11:**  There are also other agents for consideration with good emerging evidence e.g. cilostazol  Clopidogrel 150 mg /day for stroke secondary prevention is not supported by evidence or other guidelines  **Page 15:**  We agree that the bleeding risk with ticragelor could be a challenge after stroke as many patients are older and frail with comorbidities | **Specific comments on the document:**  Cilostazol is not licenced in for stroke in the UK and is mainly used for lacunar stroke in some Asian countries. The LACI-3 study is currently underway in the UK and is investigating the effectiveness of Cilostazol in lacunar stroke and small vessel disease but not large vessel strokes  The recommendation for the 150 mg/die of clopidogrel has been removed from the Tables reporting clinical actions based on genotype. It is discussed Section 7.4.  This is inconsistent with prior comments about using drugs without genetic testing, i.e ticagrelor. In those patients with a higher risk of bleeding, clopidogrel can be used in the relevant groups, and other drugs considered in those where ticagrelor considered to pose too high a risk for bleeding. | No  Yes, Tables 5-7 and Box 1-3, and Section 7.4  No |  |
|  | One potential concern that should be addressed before widespread implementation is the emerging evidence that *CYP2C19* genotype data does not necessarily correspond directly to platelet responsiveness to clopidogrel. | The NICE diagnostics guidance (**DG59 – published 31 July 2024**) did not address specifically the use of platelet function testing for the optimisation of clopidogrel therapy. Platelet function testing is not widely used and if used it is to help identify PMs who could potentially be carriers of *CYP2C19* LOF alleles. There are of course a range of factors that affect platelet function, some are affected by environmental factors and intercurrent health status and drug exposure, like the development of platelet activating antibodies, usually with heparin therapy. These factors are temporal and context specific and others affected by stable genomic factors.  Like measuring any physiological process, platelet function testing is largely context specific and temporal and will not provide a long term (lifelong) integrated average of platelet functioning in relation to a specific drug such as clopidogrel in the same way as genotyping**.** In addition, platelet function testing has not been properly validated, different platelet tests give different results. We are not aware of any emerging evidence that *CYP2C19* genotype data does not necessarily correspond directly to platelet responsiveness to clopidogrel.    We have now included the need to undertake further research on platelet function testing (Section 12.3). | No |  |
| **The Association of the British Pharmaceutical Industry (ABPI)** | Consulting the SmPC and information in the SmPC, would be helpful to know whether there is a discrepancy between this guideline and the SmPCs for products available on the UK market – has anyone done that gap analysis to determine if the guideline encourages practice that is not in line with the SmPC? There is a short section on page 21 – I presume the clinical actions by genotype are not included in 4.2 for example? Would be good to be clear about this | The clinical actions proposed in the UK CERSI-PGx guidelines are not included in section 4.2 of Plavix SmPC, nor in any SmPC of the generics available in the UK. In the MHRA database, it is available a standard template for the SmPC of clopidogrel containing medicinal products. There are few generics that lack the indication in TIA/stroke. A part this, all the information, including the pharmacogenetic annotations, are in line with the originator. | Yes, Section 10.1 |  |
|  | A table lists the authorised 4.1 indications – below this is a statement that says It is important to note that routine prescribing of clopidogrel has changed over time within clinical practice because of various factors…. I am not sure what this sentence is trying to achieve? Are we saying that the licensed indications no longer reflect clinical practice? some prescribing is off label? | This sentence aimed to clarify that the clinical use of clopidogrel has changed over time. For example, in the treatment of acute coronary syndrome, clopidogrel is no longer a first line choice in cardiovascular disease as already stated. In this situation we are thus recommending *CYP2C19* genotyping only when clopidogrel is the drug of choice.  In addition, clopidogrel has become a widely used therapeutic option in the long-term (often longer than 6 months) secondary prevention of atherothrombotic events in all ischaemic strokes, including major strokes. This is in line with the recommendations of the UK 2023 National Clinical Guideline for stroke, whereas the SmPC recommend the use in this indication from 7 days until less than 6 months. This is now included in the guideline. | No  Yes, Section 2 |  |
|  | Under recommended indications for testing – it says any patient should be considered for testing – but are there any tools or criteria that could help the prescriber? Do I read this as if a test is available you should test regardless of clinical situation – or if ‘*considering*’ do I consider a patient relatively low risk due to particular factors and I consider not to test even if available? Would a prescriber be considered negligent if they decided not to test when a test is available and the patient is harmed? | The wording regarding pharmacogenetic testing has been amended. The committee is aware that testing may not be readily available for all indications, and, in these situations, local practice should be followed.  The NCGS states in ***section 5.6***: For long-term prevention of vascular events in people with ischaemic stroke or TIA without paroxysmal or permanent atrial fibrillation:   - clopidogrel 75 mg daily should be the standard antithrombotic treatment; - aspirin 75 mg daily should be used for those who are unable to tolerate clopidogrel.   if a patient has a recurrent cardiovascular event on clopidogrel, clopidogrel resistance may be considered.  In terms of negligence, it is difficult to answer as it will be dependent on the individual circumstances, all of which would be difficult to cover in a guideline. | No |  |
|  | In the tables, it would be helpful to include references that support the clinical actions based on genotype, are all suggestions covered by a expert group guide or paper? | The UK CERSI-PGx recommendations for alternative treatments are based on the approved alternative drugs in the UK and on what is the UK routine practice as suggested by the clinicians, members of the writing committee. Whenever possible the UK relevant prescribing guidelines have been quoted. This sentence is now included in the guideline. | No |  |
| **The British In Vitro Diagnostic Association (BIVDA)** | No response received |  |  |  |
| **British Heart Foundation (BHF)** | No response received |  |  |  |
| **ClinPGx** | If you pursue peer-reviewed publication, one suggestion we have is to reconsider use of the term “guideline,” a term which implies many standardized procedures and detailed attribution characteristics (clinical practice guideline by the IOM/NAM or the AGREE II framework; see below for more elements of a guideline). We are aware of other health systems that have put together detailed procedures for their PGx implementation that heavily rely upon CPIC guidelines (e.g. in the Canadian health care system), and they have used the term “guidance,” perhaps to acknowledge that they don’t adhere to guideline metrics and rely upon “guidelines” to implement their clinical “guidance.”    Given that this is a guidance to be employed in your clinics, it is important to emphasize that implementation needs to constantly be updated with any relevant changes in genotype-to-phenotype translations and allele definitions. These are updated routinely in the CPIC database (which is downloadable via the API, which many clinical centers use regularly). We wondered if it might make sense to suggest to your labs to use CPIC’s allele function assignments and diplotype-to-phenotype tables as a reference, to be sure the procedure stays “evergreen.  If the intent is for a guideline, examples of elements of a “guideline” that CPIC includes:  ***Systematic evidence review***: The evidence section reads more like a narrative review. It might help to describe in more detail how articles were identified, selected, and appraised to ensure transparency and reproducibility.  ***Connection to recommendations:*** It would be useful to more clearly explain how the recommendations (dosing, testing, clinical workflow, allele selection) are derived from the evidence, and who was responsible for formulating them.  ***Grading of evidence:*** Established frameworks (e.g., GRADE) could provide a transparent way to evaluate the quality and strength of the evidence supporting recommendations.  ***Authorship and expertise:*** Consider clarifying who the authors are, their relevant expertise, and how conflicts of interest were managed.  ***Methods and peer review:*** Outlining the methodology, stakeholder engagement, and peer review process would increase confidence in the rigor and trustworthiness of the document.  ***Additional Specific Points***:   1. Clarify allele testing recommendations, including challenges around *1 and default alleles. 2. If AMP is the primary source for alleles to test, it may help to state that explicitly and explain why these standards were selected. 3. Include explanation of how alleles are mapped to metabolizer status, since recommendations are given at the phenotype level. E.g., the section entitled “Clinical action based on genotype” present recommendations by metabolizer status. Essentially, you may want to consider a direct connection between the alleles to test section with the recommendation phenotypes or refer to the CPIC tables. 4. Address why CAD and PAD lack rapid metabolizer recommendations in the provided tables. 5. Table 4 contains UK recommendations for likely IMs/likely PMs, but those metabolizer groups are not shown in the previous Clinical action tables or discussed in the UK sections.   We hope this feedback is helpful as you refine the document. Please let us know if you’d like to discuss further. | The UK CERSI-PGx guidelines are developed by multidisciplinary teams, including experts in clinical pharmacology, pharmacology and pharmacogenomics; clinicians from different specialities, who are the main prescribers of the specific drug; experts in pharmacy, genomic medicine and health economics. When necessary, clinical nurses are involved. The guidelines are developed for clinical use in the NHS across the four Nations of the UK. Therefore, at least one representative of the devolved Nations in addition to England is included in the guideline writing committee.  A comprehensive literature review was conducted on the three main areas of clinical use of clopidogrel, integrating information provided by the latest CPIC guideline published in 2022 and recommendations from the DPWG, available through the ClinPGx database. We also consulted other guidelines/guidance produced by specialist societies and by NICE (a few members of our committee had also served on the NICE committee which produced the NICE Diagnostics guidance DG59 on CYP2C19 genotyping after transient ischaemic attack and ischaemic stroke). In the Evidence Overview section, a summary of the most relevant evidence is provided. We included data from recent randomised clinical trials and meta-analyses. An extensive literature review was performed on specific topics, including the use of 150 mg once daily of clopidogrel in secondary cardiovascular and cerebrovascular prevention and for the health economic evaluation, prioritizing studies relevant to the UK NHS when available. Section 7 ‘Clinical actions based on genotype’ contains prescribing recommendations for alternative treatments that are based on the alternative drugs available in the UK and on what is the UK routine practice as suggested by the clinical members of the writing committee. Whenever possible relevant UK prescribing guidelines have been quoted. The full methodology is published in the Supplementary section of each guideline so that readers and users are aware of the whole development process and the meaning behind the term ‘guideline’. In conclusion. the UK CERSI-PGx guideline on the *CYP2C19* genotype testing for clopidogrel is grounded on the latest evidence in the field, although it cannot account for all individual factors relevant to patient care. Therefore, prescribers must conduct thorough assessment of each patient's response profile, ensuring that therapy is optimised to maximise benefits while minimising potential harms.  This information is now provided in the guideline  The UK CERSI-PGx has not undertaken a systematic literature review considering and acknowledging the excellent work carried out over the past 15-20 years by other international consortia, like the CPIC and the DPWG. In the UK CERSI-PGx guidelines, data from recent relevant randomised clinical trials and meta-analyses are reported referring the users to other relevant sources of information. The UK CERSI-PGx is aware of the rapidly evolving nature of the evidence, therefore if any new trial/metanalysis will come available in the future this information will be timely shared through the UK-CERSI PGx website (in development) and periodical updates of the guidelines will be provided.  All the information included in the guidelines were discussed with the experts included in the writing committee through monthly online meetings. Their feedback was collected and inputted in the text of the guideline. Details on the writing committee are provided in the Supplementary Material. Please consider that this is a multidisciplinary group, including not only experts in clinical pharmacology and pharmacy, but clinicians from the relevant disciplines using clopidogrel as well as general practitioners, experts in genomics and health economics.  As reported above, the UK CERSI-PGx recommendations for alternative treatments are based on the approved alternative drugs in the UK and on what is the UK routine practice as suggested by the clinicians, members of the writing committee. Whenever possible the UK relevant prescribing guidelines have been quoted. We have not graded the evidence, and highlighted the grading provided by CPIC and DPWG. Our recommendations are consistent with those of CPIC and DPWG, but where they are not, we have undertaken further literature reviews, and highlighted the difference in our recommendation.  Information about the authorship, expertise and COI is included in the publication.  Full methodology has been published in the Supplementary Material. It is worth noting that a final draft of the guideline was sent out for consultations to regulatory agencies, relevant speciality societies and professional networks. Comments to the guideline together with the UK CERSI-PGx responses are collated in the present Table for transparency on the entire process. In addition, the guideline will also undergo peer review by the journal.   1. The definition of allele *1 has been included. 2. This is now clearly indicated in the methodology of the guideline 3. For tier 1 variants links between genotype and phenotype are provided in Table 1. For the genotype-to-phenotype translations and allele definitions we refer the readers to the allele function assignments provided by the CPIC and the Diplotype-Phenotype table available through the ClinPGx database. The onus of translation from genotype to phenotype will be on the laboratory. In the revised version, we amended the title in the Tables 5-7 and Annexes as it follows: ***Recommended clinical actions based on the pharmacogenetic test results*** in the different indications. 4. This was a typo and has been amended. 5. The definition of “likely IM” and “likely PM” appears in the 2022 update of the CPIC guidelines (**Lee CR et al. Clin Pharmacol Ther. 2022. doi: 10.1002/cpt.2526**). It was referred to the detection of the *CYP2C19*9* allelic variant, with the following explanatory note: ‘There are limited data to characterize the function of decreased function alleles’. Table 4 (renumbered as Table 8 in the revised version of the UK CERSI-PGx guideline) includes a synthetic comparative presentation of the recommendations by different international pharmacogenetics guidelines, thus it was necessary to include the “likely IM” and “likely PM” phenotypes. This is explained in the note ‘l’ to current Table 8. The UK CERSI-PGx is not aware of laboratories that actually provide these pharmacogenetic results, but if this occurs recommendations for the IM and PM phenotype should be followed.   We value the feedback received and it has helped refine the document. | No  Yes, Section 6  No  No  No  Yes, information provided.  No  Yes, Section 6  Methodology  Yes, Tables 5-7 and Boxes 1-3  Yes, Tables 6- 7 and Boxes 2-3  Note ‘l’ to Table 8 |  |
| **The Dutch Pharmacogenetics Working Group (DPWG)** | There is a listing of the allelic frequency for the different alleles (e.g. Table 3). Whereas we in the field are quite used to work with allelic frequencies, would clinicians not be better informed when the percentage of UM, RM, NM, IM and PMs are mentioned? Like for example “Caucasian population: 2-3% PM, 20% IM, 70% NM, 6% UM)” to have a better feel of what is the chance of their patient being in either group? It would require an extra table but this is perhaps the info a clinician may want to have. | As suggested an additional Table including the frequencies of the different phenotypes has been added to the guideline (current Table 4) | Yes, Table 4 |  |
|  | The mentioning of  “likely IM” and “likely PM” (Table 4), I was a bit surprised… Which labs do report this and why? Is this from Mayo Clinics perhaps? Seems I would not be very happy as a clinician to receive such an outcome…. Technically, every heterozygote LOF CYP2C19 could be a potentially/likely PM because of missing a rare variant when using array based techniques. And every NM could be a potentially  IM or even PM… I see that these are indeed current therapeutic recommendations but  from my perspective I  was wondering whether this contributes to clarity (or just raise questions). But perhaps I missed in the manuscript on when “likely IM” and “likely PM” are being given as test result…? | The definition of “likely IM” and “likely PM” appears in the 2022 update of the CPIC guidelines (**Lee CR et al. Clin Pharmacol Ther. 2022. doi: 10.1002/cpt.2526**). It was referred to the detection of the *CYP2C19*9* allelic variant, with the following explanatory note: ‘There are limited data to characterize the function of decreased function alleles’. Table 4 (renumbered as Table 8 in the revised version of the UK CERSI-PGx guideline) includes a synthetic comparative presentation of the recommendations by different international pharmacogenetics guidelines, thus it was necessary to include the “likely IM” and “likely PM” phenotypes. This is explained in the note ‘l’ to current Table 8. The UK CERSI-PGx is not aware of laboratories that actually provide these pharmacogenetic results, but if this occurs recommendations for the IM and PM phenotype should be followed. | Note ‘l’ to Table 8 |  |
|  | Just as note: as DPWG we do not (yet) report Rapid Metabolizer (we have grouped this with NM), but this may be changed in the future | This information provided by Prof. Ron van Schaik as a personal communication, has been included in the footnote ‘g’ to the current Table 8. | Yes, Table 8 |  |
|  | One might get the impression that ticagrelor has never been assessed by a medicines agency and is therefore off label. However, CHMP of EMA assessed the benefit – risk to be negative for ticagrelor in Stroke/TIA and MAH withdrew the application: see EMA website. The Thales study was critical in this. | Changes have been made to reflect this in the regulatory section | Yes, Section 10.1 |  |
|  | The CERSI-PGx Guideline and DPWG guideline are aligned on 150 mg clopidogrel and provide similar recommendations i.e. “The use of clopidogrel 150 mg/day requires an individualised decision based on personal bleeding risk and drug efficacy considerations. However, it may offer a suitable antiplatelet strategy in some patients”. The text of the next paragraph on the use of 150 mg clopidogrel is phrased in a way that suggest a contradiction. | We have amended our recommendation regarding the use of 150 mg once daily of clopidogrel following comments from the clinical community. The text in the section 7.4 “Use of alternatively dosed clopidogrel in CYP2C19 intermediate metabolisers”, has been modified. | Yes, Section 7.4. |  |
|  | PharmGKB’s citation of DPWG recommendations is not completely up to date. I have added the current recommendations below. Besides prasugrel, also ticagrelor has a higher bleeding risk than clopidogrel. This has been deleted in the current recommendation. Furthermore, the clinical implication score is only applicable to PCI and stroke/TIA patients. | Thank you for this clarification. We have amended the wording as per your suggestions. | Yes, Section 8.2 |  |
| **Association for Clinical Genomic Science (ACGS**) | Overall the draft CERSI guidelines look very comprehensive and will become a valuable reference for clinical practice. Please see our specific comments below.     - **Lab test cost** - NICE guidelines state £44 to £139; the CERSI guidelines only mention the lower £44 figure but do qualify, stating with batching on MassArray. However, it is hard to know what the real cost of testing is without knowing the numbers per batch etc, as this has a significant impact on costs.  Therefore it might be useful for the CERSI guidelines to state the same range as quoted by NICE, to ensure both consistency and transparency. - **Alleles to be tested**- NICE does not really give guidance on which variants should be tested, but does say which variants the POC devices test for. The CERSI guidelines refer to Tier 1 and Tier 2 variants, as per the AMP guidelines (Pratt et al, 2018), but there is no clear explanation of what is meant by these Tiers. The CERSI guidelines highlight three alleles in the Tier 2 list, as being relevant to the UK population, and state these “should be considered” for inclusion in a genotyping panel. Therefore, rather than just referring to the AMP Tiers, it would be preferable for the CERSI guidelines to specifically list the alleles that should be tested for in the UK, to avoid any ambiguity. The NHSE National Genomic Test Directory has not yet specified which alleles should be tested for, therefore publication of these CERSI guidelines offers an opportunity to explicitly state which alleles should be tested. This would ensure consistency of testing across laboratories. Otherwise, the phrase “should be considered” is open to different interpretations, potentially leading to uncertainty about what is actually required and different levels of testing in different laboratories.      - **Variant Nomenclature -**The CERSI guidelines only refer to CYP2C19 alleles by the Star allele nomenclature. This could be confusing, as the Star alleles for many pharmacogenomic genes are not associated with single gene variants, but actually represent haplotypes. The CYP2C19*2 allele for example, is actually defined by two sequence variants, 12662A>G and 19154G>A. Since this is the first CERSI guideline for pharmacogenomics, it should set a precedent and ensure absolute clarity about which sequence variants are being referred to, through the use of HGVS nomenclature which defines alleles at the sequence level.  NHS Genomics labs have been using HGVS nomenclature in their clinical reports for many years, as this is deemed preferable and more reliable than alternative systems, and this should continue for pharmacogenomic reporting. Star alleles are important for PGx interpretation, so both nomenclatures are likely to be quoted on laboratory reports. To facilitate this and avoid any ambiguity about alleles, it would be helpful for the CERSI guidelines to include HGVS nomenclature as well as the star allele nomenclature. This could be documented in tabular format, showing: star allele; dbSNP rs reference number; HGVS nomenclature using the gene’s coding reference sequence NM_000769.4; HGVS nomenclature using genomic coordinates | Thank you for the positive comments and support.   - The source of £44 is based on NICE's response to stakeholder comments: *"We have explored a “high efficiency” lab-test scenario where lab tests were assumed to be processed in batches of 55 tests per batch. A batch of 55 was chosen assuming 100,000 tests per year and assuming 400 tests per working day. It is assumed that each of the 7 current NHS GLH laboratories would process these 400 tests each day. The cost of reagent, per test cost of the machine, and nursing costs were kept the same of the base case. When using a batch size of 55 samples the overall lab test cost used in Scenario 13 was £44."* - At present, most laboratories in the UK are providing pharmacogenetic testing for Tier 1 variants. However, considering the mixed genetic background of the UK population extended panels including other relevant variants to the UK population should be considered if available. It is beyond the remit of this guideline to suggest a specific test including specific sets of variants.   The term “should be considered” has been removed, where appropriate.   - The HGVS nomenclature has been included in brackets for each allele. The source of this information is the ClinPGx website at the following link <https://www.clinpgx.org/gene/PA124/haplotype> | Yes, Section 9.1  No  Yes, Section 6 |  |
| **Association for Molecular Pathology (AMP)** | **Reviewer Expertise**: board certified clinical laboratory directors with significant expertise in molecular diagnostics, experience with various pharmacogenomics testing methodologies, and understanding of the clinical utility of PGx testing. Guideline development and methodology.  **Methodology**  ***Manuscript Type***  Clinical practice guideline using consensus methodology  ***Guideline Reporting Elements***  The reviewers have indicated the following AGREE Guideline Reporting Checklist domains below that have been **satisfactorily addressed**:   - SCOPE AND PURPOSE (objectives, questions, population) - CLARITY OF PRESENTATION (specific & unambiguous recommendations, management options, identifiable key recommendations) - APPLICABILITY (facilitators and barriers, implementation advice/tools, resource implications, monitoring and/or auditing criteria)   The following domains have been partially addressed:   - STAKEHOLDER INVOLVEMENT (group membership, target population preferences/views, target users) - RIGOUR OF DEVELOPMENT (search methods, evidence selection criteria, strengths & limitations of evidence, formulation of recommendations, consideration of harms & benefits, link between recommendations & evidence, external review, updating procedure)   **Scientific content**   - Thorough, practical and clear - Content was clearly communicated   AMP is aware that the comments below were returned to the author(s) as individual feedback from Dr. Pratt (Co-chair, AMP PGx Working Group):   - Page 9 – recommend that make clear that a *1 is no variant detected and is not actually tested. - Page 9-10 – recommend to include HGVS nomenclature, in parens? For each star allele as allele definitions can/may change. There will be an update to *2 and *35. - This is minor comment and more a pet peeve of mine that while the use of “carry/carrying/carriage" is pervasive in PGx literature as a geneticist, carriers do not have clinical symptoms (think CF carrier).  Individuals who have a PGx variant can have clinical impact when exposed to a medication.  I prefer the “have/having” or other appropriate terms.   During the AMP review process, a reviewer provided the following feedback: “I agree with all of [Dr. Pratt’s] comments. I think that each of these should be clarified in the manuscript. I do not have any additional comments.” For this reason, they are included verbatim.  **Organization**   - Very well organized and easy to follow - Well organized   **Argumentation**   - Very strong - Well executed   **Evidence**   - Very strong - Relevant, recent evidence cited to support arguments   **Language and Style**   - Very clear, concise and coherent - Well written   **Strengths**   - Very well organized, clear, reasoned and practical - Focus on implementation guides was well done   **Areas for Improvement**   - Description of the working group and methods by which guidelines were developed - COI disclosure / management   **Recommendations**  Include Methods section that more explicitly addresses AGREE stakeholder involvement and rigour of development domains   - Please provide additional context about the group of authors, funding sources, methods for literature review and methods for guideline development. - Because COI disclosures / management were not provided, it was difficult to determine if there were potential perceived and/or actual conflicts. eviewers   **Reviewer Remarks**   - Thank you for this excellent evidence-based guideline for testing and clinical care. This guideline will improve care for a large and complex patient population - The additional provider-focused pieces at the end were very well done. Thank you for the opportunity to review. | - STAKEHOLDER INVOLVEMENT – The authorship of this guideline is included together with their areas of expertise and conflicts of interest in the Supplementary Material. The guideline is directed to the main prescribers of clopidogrel, although we expect that other stakeholder may be interested, as for example industry developing pharmacogenetic tests and clinical decision support system. - RIGOUR OF DEVELOPMENT – The methodology employed by the CERSI is included in the Supplementary Material. - As suggested the *CYP2C19**1 allele has been removed from the list of tier 1 variants. - As suggested the HGVS nomenclature has been included in brackets for each allele. The source of this information is the ClinPGx website at the following link <https://www.clinpgx.org/gene/PA124/haplotype> - As suggested the words “carry/carrying/carriage" have been removed from the text   Information on the working group and COI disclosure are included in the published guideline as supplement. Description of the methodology is also included as Supplementary material.  As stated above, this information is now included in the Supplementary Material.  Thank you for the positive comments and support. | No  No  Yes, Section 6  Yes, Section 6  Yes, all document  Yes, Suppl. Material  Yes, Suppl. Material |  |
| **UK-Industry PGx Network (UK-IPN)** | Page 1 – Including a contents page could enhance the usability and navigation of the document, especially for readers seeking specific sections. | The guideline will be submitted to the British Journal of Clinical Pharmacology and will be formatted according to the journal style. | No |  |
|  | Page 2 Executive summary - It may be helpful to include the intended audience for this guideline at the beginning of the summary, to provide clarity and context for readers from the outset. | The guideline is directed to the main prescribers of clopidogrel, although we expect that other stakeholder may be interested, as for example industry developing pharmacogenetic tests and clinical decision support system. | No |  |
|  | Page 2 – ‘regardless of the underlying indication’-  Regarding the practical implementation of this, are CYP2C19 tests currently commissioned for use across all relevant clinical indications, allowing clinicians to access them for their patients? Or are they presently limited to the NICE-recommended indication following stroke or TIA? If the latter then, how will this be implemented if the tests are not commissioned for all indications? | At present, *CYP2C19* pharmacogenetics testing has not been commissioned by the NHS for all licensed indications of clopidogrel. After the publication of the NICE diagnostics guidance (**DG59 – published 31 July 2024**), different implementation programmes across the country have been developed to provide pharmacogenetic testing in the stroke/TIA clinical setting. The aim of the UK CERSI-PGx guideline is to raise awareness around the diminished efficacy of clopidogrel in CYP2C19 intermediate and poor metabolisers. This occurs in any clinical setting in which clopidogrel is used. Therefore, pharmacogenetic testing should be extended to all licenced indications whenever clopidogrel is the drug of choice. | No |  |
|  | Page 2 –‘grounded in the latest evidence’ - To help future-proof this guidance, it may be advisable to include the dates of your systematic evidence search, as referring to the 'latest' evidence can quickly become outdated. Including the search protocol in an appendix could also enhance transparency and reproducibility. | The UK CERSI-PGx has not undertaken a systematic literature review considering and acknowledging the excellent work carried out over the past 15-20 years by other international consortia, like the CPIC and the DPWG. In the UK CERSI-PGx guidelines, data from recent relevant randomised clinical trials and meta-analyses are reported referring the users to other relevant sources of information. The UK CERSI-PGx is aware of the rapidly evolving nature of the evidence, therefore if any new trial/metanalysis will come available in the future this information will be timely shared through the UK-CERSI PGx website (in development) and periodical updates of the guidelines will be provided. The methodology is included in the supplementary material. | No |  |
|  | Page 3 – Background Overview – It may be helpful to clearly outline the intended target audience for this guideline, to ensure the content is appropriately tailored to the target audience. | Not necessary | No |  |
|  | Page 4 – Evidence Overview – Was a search protocol developed to identify all relevant evidence and guidance documents? Including this—along with details of inclusion and exclusion criteria—could strengthen the credibility and transparency of the guideline.  What methodology has been used to develop this guideline? Whilst I appreciate the group are not guideline developers, as this is the first of perhaps many that may come through and also as CERSI-PGx is being established and expanding recognition it would be useful to have some process outlined and documented within this document for transparency, reproducibility and credibility. | The most recent evidence in all clinical settings have been included in more detail in the guideline, particularly data from recent randomised clinical trials and meta-analysis. The UK CERSI-PGx has not performed a systematic literature review, since has acknowledged the excellent work done in the field by other well established pharmacogenetics international consortia, namely the CPIC and the DPWG.  All the information included in the guidelines were discussed with the experts included in the writing committee through monthly online meetings. Their feedback was collected and inputted in the text of the guidelines. Details on the writing committee are provided in a separate document. Please consider that this is a multidisciplinary group, including not only experts in clinical pharmacology, pharmacology and pharmacy, but clinicians from the relevant disciplines using clopidogrel as well as general practitioners, experts in genomics and health economics. A four Nation approach has been undertaken, considering the views of representatives from the devolved nations in addition to England. The full methodology is published in the Supplementary Material of each guideline so that readers and users are aware of the whole development process. | No  No |  |
|  | **Page 8 – Existing Clinical Pathways-** Could the guidance clarify who is responsible for requesting these tests? I assume this would typically fall within secondary care due to the acute nature of presentation, but it may be helpful to make this explicit.  To support implementation and integration into clinical pathways, would it be helpful to include guidance on updating existing protocols for managing TIAs, particularly in light of the national NICE recommendation for this indication? | *CYP2C19* genotyping will largely be initiated in secondary care. A specific section on communication between secondary care and primary care is included. This was prepared with the input of two general practitioners, members of the writing committee.  Reference to the NICE diagnostics guidance (**DG59 – published 31 July 2024**) has been made throughout the document, including a specific paragraph in the REGULATORY CONSIDERATIONS section (Section 10.2). | No  No |  |
|  | **Page 9 ‘turn around time’ –** suggest - 'and minimum recommended turnaround times' If included these documents will guide required service delivery standards for industry and NHS services. | This information is based from the experience gathered in Scotland with the *CYP2C19* testing as well as during the initial phases of the pilot project of *CYP2C19* implementation in England. | No |  |
|  | **Page 11 clinical actions based on genotype -** When viewing the whole document, there is a lot of different recommendations and actions discussed. Please label headings very clearly: could this be CERSI or UK clinical actions/ recommendations based on genotype? | Harmonisation throughout the text has been performed throughout the tables. | Yes |  |
|  | **Page 11 – prescribing suggestions in table –**  'Prescribing suggestions' is a non directional phrase. The language being used in the recommendations below are directional statements.  Would this be better as 'guidance' or 'considerations''?  Ideally Industry partners should be able to use this guidance as a reference when developing their products for the UK market. This would ensure that they are using UK approved guidance statements that can be consistent across different medical devices.  To achieve this, it is anticipated that Industry partners would lift content from these statements to build a recommendation, so clear language or statements required to avoid room for confusion or interpretation. | The UK CERSI-PGx guidelines are developed by multidisciplinary teams, including experts in clinical pharmacology, pharmacology and pharmacogenomics; clinicians from different specialities, who are the main prescribers of the specific drug; experts in pharmacy, genomic medicine and health economics. When necessary, clinical nurses are involved. The guidelines are developed for clinical use in the NHS across the four Nations of the UK. Therefore, at least one representative of the devolved Nations in addition to England is included in the guideline writing committee.  For the Evidence Overview section, the UK CERSI-PGx guidelines refer to recent pharmacogenetic guidelines (when available), provided by the two main international consortia in pharmacogenetics, the Clinical Implementation Pharmacogenetics Consortium (CPIC) and the Dutch Pharmacogenetics Working Group (DPWG). These consortia have provided prescribing recommendations for several gene-drug pairs based on systematic literature reviews carried out over the last 15-20 years. In addition, CERSI-PGx perform its own literature evaluation on specific topics, for example when recommendations between CPIC and DPWG diverge. Therefore, recommendations from the UK CERSI-PGx consortium are evidence-based together with expert input from committee members who are able to highlight nuances in specific clinical settings in which a specific drug is used. The full methodology is published in the Supplementary section of each guideline so that readers and users are aware of the whole development process and the meaning behind the term ‘guideline’. In conclusion. the UK CERSI-PGx guideline on the *CYP2C19* genotype testing for clopidogrel is grounded on the latest evidence in the field, although it cannot account for all individual factors relevant to patient care. Therefore, prescribers must conduct thorough assessment of each patient's response profile, ensuring that therapy is optimised to maximise benefits while minimising potential harms. |  |  |
|  | **Page 16 Other Pharmacogenetics Guidelines -** Could this section be in a separated chapter to make sure it is clear that this is not the UK recommendation. Industry will be looking for clear guidance on wording that should be incorporated into reports used for the UK market. All this information listed closely together in the same chapter may confuse organizations wanting to lift recommendations into their reports. | This section is indeed a separate chapter. The purpose of the section has been highlighted in the revised version of the guidelines. | Yes, Section 8 |  |
|  | **Page 18 Table 4 –** If the CERSI recommendation is to be used as the primary source of information in the UK - this should be clearly stated in the table, positioned as the first column, with the column colour standing out ahead of other recommendations. | The main purpose of this table (Table 8 in the revised version) is to compare the UK CERSI-PGx recommendations to the recommendations provided by other international guidelines in a synthetic manner. This is the reasons why the UK CERSI-PGx recommendations are included (in an abbreviated format) in the last column. The users should refer to Tables 5-7 in the section 7, CLINICAL ACTION BASED ON GENOTYPE, and in the Boxes 1-3 included at the end of the document. | No |  |
|  | Page 28, Annexe A B C - Annex A.B & C  Please can you formally title these as UK CERSI recommendations.  There are so many recommendations in the document that the title needs to be clear, so that when it is lifted the source and content are easily identifiable. For example NICE guidance quotes guidance review number, and title etc... They have recommendation summary easily accessible at the top of the guideline document. Every section is headed and numbered.  If keeping the full guidance in the annex then a link or reference directing people to the annex would be helpful  Most patient facing users will be needing quick access to the guidance, the scientific justification is necessary but will be used less. | These have now been relabelled as Boxes 1-3.  The wording ‘(developed by the UK CERSI-PGx)’ has been added  The boxes have been formatted as a single page document to summarise the recommendations. Moreover, in the executive Summary, the following sentence is included: ‘Summary guidance on a page is provided for each of the indications in boxes 1-3’. This is to direct the reader to the one-page summary for each licensed indication of clopidogrel.  The guideline will be formatted as per journal style  Boxes 1-3 will be printed according to journal style. | Yes, Boxes 1-3  No  No  Yes, Executive summary |  |
